# Synthesis, characterization, antimicrobial, cytotoxic and carbonic anhydrase inhibition activities of multifunctional pyrazolo-1,2-benzothiazine acetamides

**DOI:** 10.3762/bjoc.21.25

**Published:** 2025-02-12

**Authors:** Ayesha Saeed, Shahana Ehsan, Muhammad Zia-ur-Rehman, Erin M Marshall, Sandra Loesgen, Abdus Saleem, Simone Giovannuzzi, Claudiu T Supuran

**Affiliations:** 1 Department of Chemistry, Lahore College for Women University, Jail Road, Lahore 54000, Pakistan; 2 Applied Chemistry Research Centre, PCSIR Laboratories Complex, Lahore 54600, Pakistan; 3 Whitney Laboratory for Marine Bioscience, University of Florida, St. Augustine, Florida 32080, USA; 4 Govt. Shalimar Graduate College, Baghbanpura, Lahore 54920, Pakistan; 5 Neurofarba Department, Sezione di Scienze Farmaceutiche e Nutraceutiche, University of Florence, 50019 Florence, Italy

**Keywords:** acetamides, antibiotics, antimicrobial, benzothiazines, *Staphylococcus aureus*

## Abstract

The advent of antibiotic resistance in microorganisms requires the discovery and synthesis of novel antibiotics. At the same time, human pathogens are contributing to chronic and persistent inflammation. Motivated by these two concerning issues, new antibiotic drug candidates are synthesized by incorporation of benzothiazine, pyrazole, and amide moieties in a new scaffold to create multifunctional derivatives of pyrazolo-1,2-benzothiazine. The presented compounds have been synthesized and analyzed using spectroscopic and spectrometric techniques including FTIR, HRMS, ^1^H and ^13^C NMR spectroscopy. All compounds were tested against five human microbial strains including three different strains of *Staphylococcus aureus* (ATCC 25923, ATCC BAA-41, and ATCC BAA-44), *Escherichia coli* (ATCC 8739), and *Candida albicans* (ATCC 90027) to evaluate their antibiotic potential. The results showed that out of fourteen synthesized compounds, **7b** (MIC_90_ = 16 μg/mL) and **7h** (MIC_90_ = 8.0 μg/mL) exhibited potent antibiotic activity against different strains of *S. aureus* (susceptible, methicillin-resistant, and multidrug-resistant). Cytotoxic studies against the human colon cancer mammalian cell line HCT-116 (ATCC CCL-247) revealed that only compound **7l** inhibited cell viability, while the rest of the compounds including **7b** and **7h** showed no significant decrease in mammalian cell viability. Results of human carbonic anhydrase (hCA) inhibition assays discovered that monoalkylated derivatives have low to negligible inhibition potential but dialkylated ones have no inhibition potential at all for directed CAs (I, II, IX, and XII). From the low inhibiting compounds, **7b** showed the highest inhibition potential with a minimum *K*_i_ value of 72.9 μM. In light of the above findings, these newly prepared scaffolds are valuable additions to the class of pyrazolo-1,2-benzothiazine antibiotics with selective antistaphylococcal activity.

## Introduction

The global crisis of antibiotic resistance has been progressing for decades. Reasons behind this issue include over-prescribing of FDA-approved drugs, agricultural use, rapid resistance development, and declining rates of novel antibiotic discovery [1]. In fact, the rate of discovery of new classes of antibiotics is low compared to the development of resistance [2]. Innovative scaffolds for novel antibiotic drug candidates are required to create new methods for safe and effective treatments. Chemically designed drug leads can complement naturally found antibiotics and may engage multiple modes of action and might have a longer lifetime before resistance evolves [3].

Complex chemical scaffolds with more than one protein-engaging functionality in a single molecule are advantageous for selectivity. This concept of synergistic compounds and complex chemical interactions helps to boost biological activity and prolongs the emergence of resistance in pathogens [4]. Notable previous efforts include the synthesis of benzothiazine scaffolds connected to other heterocyclic moieties such as piperazine [5], triazole [6–7], hydantoin [8], and pyrazole moieties [9–10]. Very few examples of pyrazolobenzothiazines presenting an amide moiety are published. This work covers the synthesis of synergistic scaffolds containing biologically active 1,2-benzothiazine, pyrazole, and amide moieties.

1,2-Benzothiazines are chemically gifted drug candidates. Since one of the very first synthesis in 1956 [11], these scaffolds have proved themselves as versatile and biologically active useful candidates. NSAIDs (non-steroidal anti-inflammatory drugs) currently approved like piroxicam [12], meloxicam [13], and other derivatives of these two are well known for their activities [5]. 1,2-Benzothiazines are highly bioactive moieties and have been explored as antibacterial [14–15], antiviral [16], antifungal [14,17], antirheumatic [18], antiparasitic [19], insecticidal [20], antituberculous [21–24], antitumor [25], antileukemic [26], antidiabetic [27], and antifertility therapeutics [28]. Some 1,2-benzothiazine derivatives have been explored in the treatment of autoimmune diseases [18], osteoarthritis [29], hemorrhage [25], and cardiac diseases [30].

Pyrazole moieties have a wide spectrum of pharmacological efficiencies. One known example is celecoxib^®^, a clinically used anti-inflammatory and COX-2 enzyme-inhibiting drug [31]. In some cases, inclusion of a pyrazole moiety was beneficial to optimize therapeutic activity of different analogs with lesser adverse effects [32].

Amide linkages are a common drug feature that comprises about 25% of the most prescribed and vended medication [33]. Mimicking biologically relevant structural features of proteins and enzymes, makes them a suitable linker unit [34]. The leading example is paracetamol highlighting the utility of an amide containing scaffold in medicinal chemistry.

A series of novel scaffolds adjoining benzothiazine with a pyrazole moiety has been reported with antioxidant activity and antibacterial potential [9]. Pyrazolobenzothiazines have also been synthesized and explored as p38α MAPK inhibitors [35]. In 2015, Sabatini and co-workers synthesized a novel series of pyrazolobenzothiazines and identified as new generation anti-inflammatory agents. Two of these compounds are reported with an IC_50_ of 0.5 µM to inhibit p38α MAPK and 0.5 µM for TNF-α [10]. Pyrazolobenzothiazines containing a triazole moiety have also been studied as potential antibacterial drugs [7].

**Figure 1:**
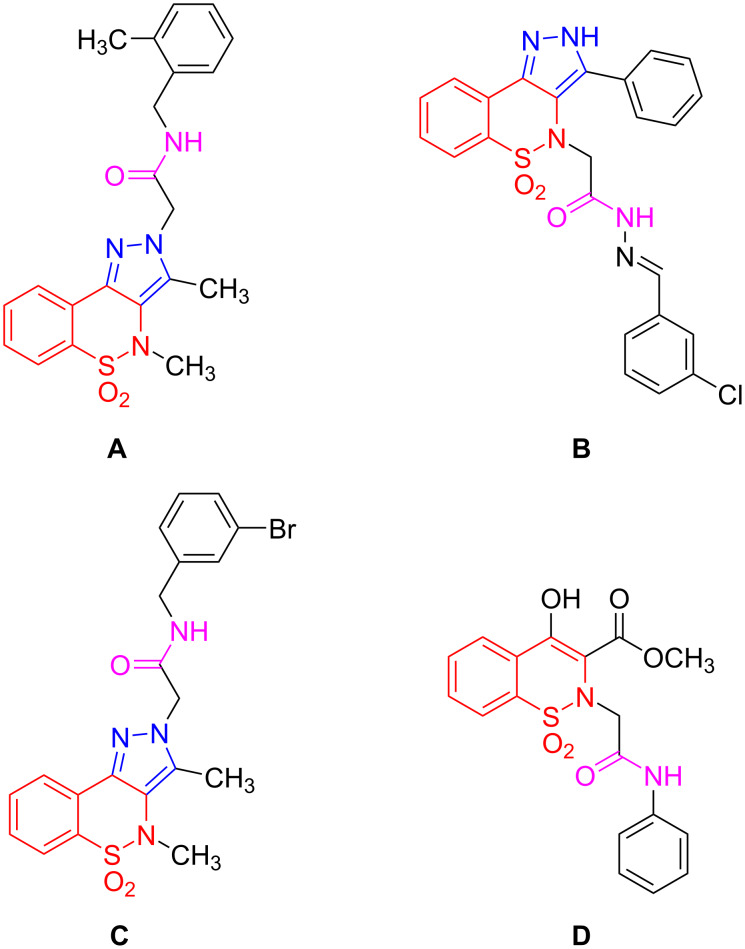
An overview of previously synthesized 1,2-benzothiazines [36–39].

Other applications of *N*-substituted benzyl/phenyl acetamide pyrazolobenzothiazines include superoxide anion and DPPH radical scavenging assays [36] (Figure 1A). In continuation of this work, these derivatives have also been investigated for their remarkable anti-HIV-1 activity [37] (Figure 1B). The same research group reported the synthesis of acetamide derivatives of pyrazolo-1,2-benzothiazines as anticancer drugs using a borane-THF complex as the amide coupling agent with thionyl chloride [38] (Figure 1C). Recently, benzothiazine scaffolds of phenyl acetamides were synthesized as potent inhibitors for ureolytic infections [39] (Figure 1D).

Saccharine (a sweetener) was reported to be a potent carbonic anhydrase inhibitor (CAI) by Klebe’s research group in 2007 [40]. A literature survey revealed that various heterocyclic compounds especially benzenesulfonamides can be best CAIs [41–43]. These CAIs are used to treat different pathological conditions like epilepsy [44], glaucoma [45], diabesity [46], obesity [47], neuropathic pain [48], cancer [41], hypertension (as diuretics) [41], and arthritis [49].

This article introduces a new approach to synthesize the targeted scaffolds from commercially available saccharine without using amide coupling agents to minimize the cost. This work is the first attempt to put pyrazolo-1,2-benzothiazine derivatives into a carbonic anhydrase inhibition assessment test. The design of the present research work is emphasized by all mentioned pharmaceutical contributions of benzothiazines and adjoining scaffolds. After synthesis following characterization, these new pyrazolobenzothiazine scaffolds were evaluated for their antimicrobial, cytotoxic, and human carbonic anhydrase (hCA) inhibition potential.

## Results and Discussion

### Chemistry

**Scheme 1:**
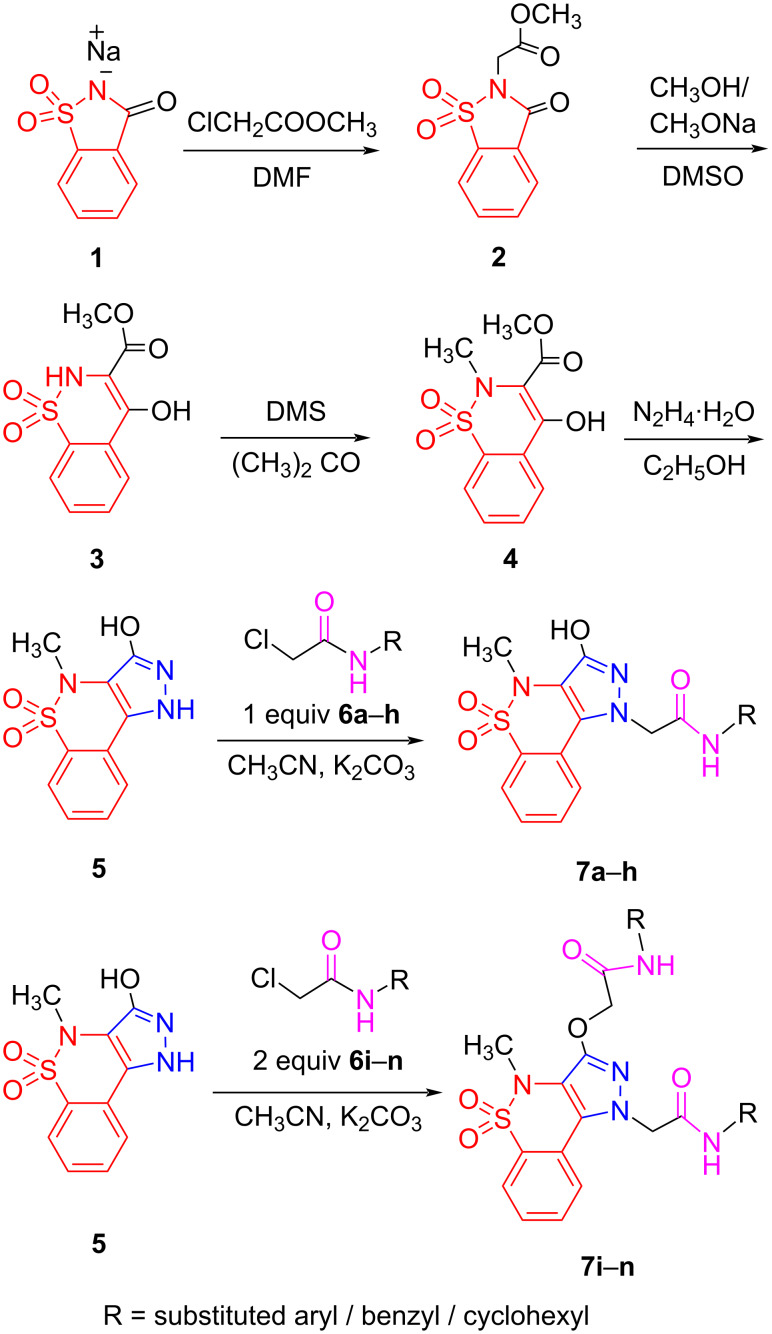
General scheme for the synthesis of pyrazolo-1,2-benzothiazine-*N*-aryl/benzyl/cyclohexylacetamide.

The targeted compounds were synthesized using the general scheme shown in Scheme 1. The ester group was introduced at the nitrogen of saccharine sodium (**1**) using dry DMF. The five-membered ring of this esterified benzisothiazole **2** was then expanded to form a six-membered cycle via a ring-expansion reaction in anhydrous conditions. In this reaction, the benzisothiazole scaffold was converted into a benzothiazine backbone **3** followed by *N*-methylation to obtain the derivative of 1,2-benzothiazine-3-carboxylate **4**. Compound **4** was reacted with an excess of hydrazine monohydrate to introduce the pyrazole moiety into the benzothiazine scaffold. Subsequently, pyrazolobenzothiazine **5** was elaborated with alkylating agents **6a**–**h** to give *N-*alkylated (monoalkylated) products and with alkylating agents **6i**–**n** to give the *N-* and *O-*alkylated (dialkylated) products. The alkylation of compound **5** was controlled by the molar quantities of alkylating agents and base present. Thus, compound **5** was *N*-alkylated using equimolar quantities of the alkylating agents **6a–h** to give the respective derivatives **7a**–**h**. On the other hand, compound **5** was *N*-alkylated as well as *O*-alkylated using 2.0 equivalents of alkylating agents **6i**–**n** to yield the respective derivatives **7i–n**.

The formation of monoalkylated derivatives is based on the fact that the NH group of the pyrazole ring in **5** is more susceptible to alkylation than the OH group. However, under more basic conditions and using the alkylating agent in excess can lead to a dialkylated product. It has already been established that *N*-alkylation takes place before *O*-alkylation because the nitrogen atom is a softer nucleophile as compared to the oxygen atom [50]. Preferential *N*-alkylation (over *O*-alkylation) of 1,2-benzothiazine scaffolds has also been carried out by Ahmad and co-workers in 2014 and Szczęśniak-Sięga and companions in 2018 [37,51].

**Figure 2:**
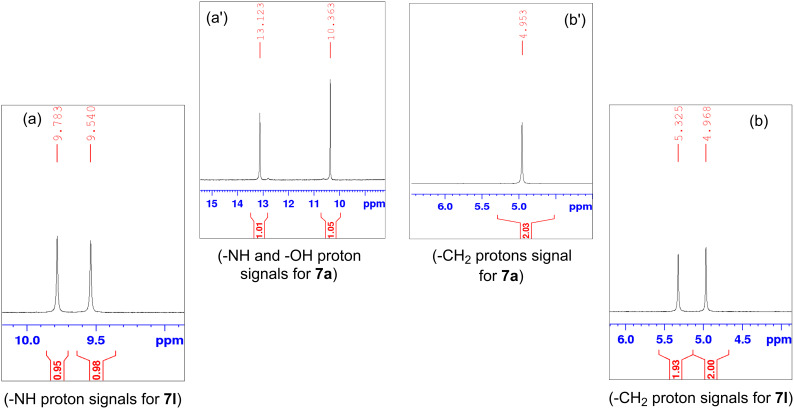
An example of contrasting ^1^H NMR signals for monoalkylated (**7a**) and dialkylated (**7l**) derivatives, (-NH and –OH = a, a’ and -CH_2_- = b, b’). Full spectra are shown in Supporting Information File 1.

Structure elucidation of all the synthesized derivatives was carried out using ^1^H, ^13^C NMR, and FTIR spectroscopy as well as HRMS. Table 1 shows the different substituents (R groups) for the synthesized compounds **7a**–**n**. The ^1^H NMR spectra of compounds **7a**–**h** showed one distinct singlet at 4.92 to 5.12 ppm for the two protons of one methylene group (Figure 2b’), a singlet for the NH proton in the range of 10.03 to 10.40 ppm, and another singlet for the OH proton in the range of 13.00 to 13.20 ppm (Figure 2a’). The presence of a singlet near 13 ppm for the OH proton confirms that the compounds **7a**–**h** are *N-*alkylated. The ^1^H NMR spectra of compounds **7i**–**n** showed two distinct singlets from 4.90 to 5.30 ppm for the two protons of each methylene group (Figure 2b). These compounds showed two individual singlets each for one NH proton in the range of 9.54–11.06 ppm (Figure 2a). The absence of signals near 13 ppm for OH protons confirms the formation of *N-* and *O-*alkylated compounds **7i**–**n**. Both sets of compounds **7a**–**h** and **7i**–**n** shared a singlet for the *N*-methyl protons in the range of 3.07 to 3.10 ppm. Signals for aromatic protons were recorded from 6.70 to 8.19 ppm depending upon the extent of shielding/deshielding.

**Table 1:** Different *N*-aryl/benzyl/cyclohexyl groups.

Comp.	R group	Comp.	R group	Comp.	R group

**7a**	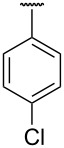	**7f**	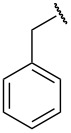	**7k**	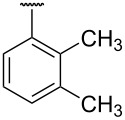
**7b**	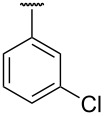	**7g**	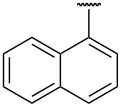	**7l**	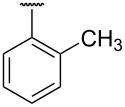
**7c**	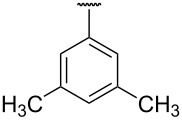	**7h**	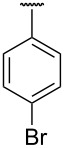	**7m**	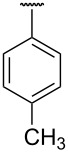
**7d**	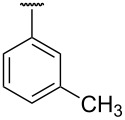	**7i**	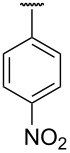	**7n**	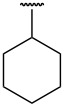
**7e**	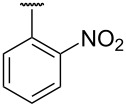	**7j**	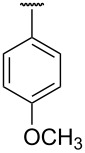		

The ^13^C NMR spectra exhibited distinct signals for the synthesized compounds **7a**–**h**: one methylene at 67.7–68.0 ppm, one *N*-methyl at 38.1–38.2 ppm, and one carbonyl carbon signal at 166.5–167.6 ppm. Also for compounds **7i**–**n**, distinct ^13^C NMR chemical shifts were observed: two methylene carbon resonances at 54.7–68.2 ppm, one *N*-methyl at 37.9–38.0 ppm, and two carbonyl carbon signals at 165.2–166.7 ppm. Aromatic carbon chemical shifts for both sets of derivatives **7a**–**h** and **7i**–**n** were found in the range of 109.1–156.0 ppm.

### In vitro antimicrobial and SAR analysis

Microbroth single dose antimicrobial assays were performed against different pathogens including susceptible *Staphylococcus aureus* (ATCC 25923), *Escherichia coli* (ATCC 8739), and *Candida albicans* (ATCC 90027). Pathogens were treated with 125 μg/mL of tested compounds in duplicate. Two compounds, **7b** and **7h**, showed significant antibacterial activity against the susceptible strain of *S. aureus*. Moreover, some compounds showed moderate to weak antimicrobial efficiency against *E. coli*, and *C. albicans* (see Figure S43 in Supporting Information File 1). These experiments showed that compounds **7b** and **7h** have potential antistaphylococcal activity.

Microbroth dilution assays [52] were performed for the determination of MIC (minimum inhibitory concentration, MIC_90_) values for compounds **7b** and **7h** against three different strains of *Staphylococcus aureus*: susceptible (ATCC 25923), methicillin-resistant (ATCC BAA-41), and multidrug-resistant (ATCC BAA-44). Table 2 shows the MIC_90_ values for the two halogenated compounds against different strains of *S. aureus*. The MIC_90_ values for compound **7b** were found to be 16 μg/mL and the MIC_90_ values for compound **7h** were found to be 8 μg/mL for all *S. aureus* strains tested.

**Table 2:** Experimental data of microbroth dilution assays against different strains of *S. aureus.*^a^

Antibiotic agent	MIC_90_ (μg/mL)		

	SA	MRSA	MDRSA

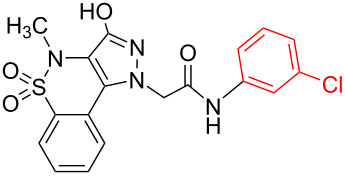 **7b**	16	16	16
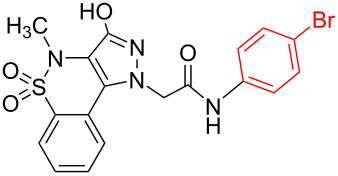 **7h**	8	8	8
positive control^a^	<1	<1	<1

^a^MIC_90_ = Minimum inhibitory concentration for 90% growth inhibition of pathogens; susceptible *S. aureus* (SA), methicillin-resistant (MRSA), multidrug-resistant (MDRSA). SA control is kanamycin, MRSA and MDRSA control is vancomycin.

Halogenation on the benzene ring of the acetamide group showed increased activity against *S. aureus* with compound **7h** the most potent. Interestingly, the presence of a halogen, preferably a bromine substituent at the *para*-position of the acetamide group increased the antibiotic potential against *S. aureus*. A comparative analysis of the antimicrobial data between mono/dialkylated derivatives revealed that *O*-alkylation did not improve the antimicrobial potential.

### Cell viability study

Single-dose MTT assays were performed for compounds **7a–n** against the human colon cancer mammalian cell line (HCT-116, ATCC CCL-247) for evaluating their inhibition of mammalian cell metabolism [53–55]. Solutions of the tested compounds were prepared in DMSO and cancer cells were treated with 10 μM doses of compounds using the apoptosis inducing standard mensacarcin (10 μM) as a positive control [56]. Only compound **7l** decreased cell viability to 63%, while the rest of the compounds showed very low to no significant decrease in cell viability (Figure S44 in Supporting Information File 1).

### In vitro carbonic anhydrase inhibition

In vitro assessment of all targeted pyrazolobenzothiazine scaffolds for human carbonic anhydrase (hCA) inhibition was performed adopting a stopped-flow CO_2_ hydration method. The enzyme inhibition assays were carried out for four types of human CA isoforms: CA I, CA II, CA IX, and XII. The first two are cytosolic forms while the other two are transmembrane forms. Reason for the selection of these CA isoforms was to develop a comparative relationship for inhibition selectivity between CAs (IX, XII) attributed to tumour/cancer and other CAs (I, II). Non-selective inhibitors may have certain adverse effects so selective CAIs are worthier and more valuable [57]. Results of hCA inhibition assays are listed in Table 3. It was observed that the dialkylated pyrazolobenzothiazines **7i**–**n** failed to inhibit any of the four targeted hCAs. One of the monoalkylated compounds, **7g**, also appeared to be inactive for the inhibition of all four targeted hCAs. The data also revealed that compounds **7a**–**h** were found inactive for CA I type. However, some compounds from **7a** to **7h** were found to show low inhibition activity against three types of CAs (CA II, CA IX and CA XII). Among these low active compounds, the highest inhibition (*K*_i_ = 72.9 μM) was shown by compound **7b** for the CA IX type. This compound also exhibited low to negligible inhibition with *K*_i_ values of 77.4 and 90.9 μM for CA XII and CA II, respectively. The compounds with very low CA II inhibition (*K*_i_ = 90–95 μM) include **7c, 7f**, and **7h**. Again very low inhibition activity with *K*_i_ values 90 to 95 μM was exhibited by compound **7a** for CA XII and compound **7h** for CA II, CA IX, and CA XII, respectively. Slight inhibition potential with *K*_i_ values 79 to 88 μM was shown by **7a** for CA II, **7c** for CA IX, **7d** for CA II, CA IX and CA XII, and **7f** for XII. However, compound **7e** appeared to show low but selective inhibition against the two transmembrane and tumor-associated forms (CA IX and CA XII) with *K*_i_ values of 82.6 and 88.4 µM, respectively and no inhibition against CA I and CA II. Finally, none of the pyrazolobenzothiazine scaffolds showed hCA inhibition activity comparable to acetazolamide (AAZ, reference drug).

**Table 3:** Experimental data of human carbonic anhydrase (hCAs) inhibition assays adopting a stopped-flow CO_2_ hydration method.

Comp.	*K*_i_ (μM)^a^ for targeted CAs				Comp.	*K*_i_ (μM)^a^ for targeted CAs			
	**I**	**II**	**IX**	**XII**		**I**	**II**	**IX**	**XII**

**7a**	>100	84.6	>100	90.2	**7i**	>100	>100	>100	>100
**7b**	>100	90.9	**72.9**	77.4	**7j**	>100	>100	>100	>100
**7c**	>100	93.2	79.2	>100	**7k**	>100	>100	>100	>100
**7d**	>100	86.5	78.5	81.8	**7l**	>100	>100	>100	>100
**7e**	>100	>100	82.6	88.4	**7m**	>100	>100	>100	>100
**7f**	>100	90.3	>100	79.1	**7n**	>100	>100	>100	>100
**7g**	>100	>100	>100	>100	**AAZ** **^b^**	0.25	0.012	0.025	0.0057
**7h**	>100	95.2	90.8	91.7					

^a^Mean from three different assays (errors = ±5–10% of the reported value); ^b^acetazolamide = reference drug.

## Conclusion

In summary, an economical multistep synthesis approach without using any coupling reagents for the synthesis of new multifunctional pyrazolo-1,2-benzothiazines was successfully validated in this article. The synthesized compounds were characterized using various spectroscopic and spectrometric techniques (^1^H and ^13^C NMR, FTIR, HRMS). The antibiotic activity of these compounds was assessed against three different pathogens and counter-tested for inhibition of mammalian cells in vitro. Results of the microbroth single-dose assays revealed that two of the synthesized compounds have selective antibiotic activity against *S. aureus.* Subsequently, MIC_90_ values were calculated by performing microbroth dilution assays using serial dilutions of these two compounds against three different strains of *S. aureus*. Compound **7b** has a MIC_90_ of 16 μg/mL, and **7h** exhibited a MIC_90_ of 8 μg/mL against susceptible, methicillin-resistant, and multidrug-resistant *S. aureus*. So, it was observed that both of these compounds destroyed designated bacterial cells in a competitive fashion. In addition, these compounds showed very low to no inhibition of human colon cancer cell line (HCT-116), with the exception of compound **7l** which reduced the cell viability about 63% and is currently further investigated. Based on the above findings, compound **7b** and **7h** are considered antistaphylococcal drug leads with compound **7h** being the most potent.

Results of the first enzymatic inhibition analysis for pyrazolo-1,2-benzothiazine derivatives against selected isoforms of hCA (I, II, IX, XII) revealed that few of them (**7a**, **7b**, **7c**, **7d**, **7e**, **7f**, and **7h**) exhibited inhibition potential at the micromolar level against different hCA isoforms. Among these weak CA inhibitory candidates, compound **7b** appeared with highest inhibition potential and minimum *K*_i_ = 72.9 μM against CA IX. Compound **7e** emerged as most selective compound against two transmembrane and tumor/cancer-related forms (CA IX and CA XII) with *K*_i_ values of 82.6 and 88.4 µM, respectively.

## Experimental

All solvents were used after double distillation. Other chemicals involved in synthesis were procured from Sigma-Aldrich and Alfa Aesar and used without further purification. Melting temperatures of all the synthesized compounds were noted using a Gallenkamp melting point apparatus. For FTIR spectra, a Bruker Alpha spectrometer (model 200262) was used. NMR spectra were obtained using a Bruker Avance spectrometer operating at 300 and 75 MHz for ^1^H and ^13^C, respectively. HRMS analysis was performed using an Agilent 6546 LC/Q-TOF for ESI scan in the positive mode to get *m*/*z* (mass/charge) values of different molecular adducts.

### General procedures for synthesis

Compounds **2**–**4** were synthesized adopting procedures given in the literature [58–59] and compounds **6a**–**n** were synthesized using a procedure reported in the literature [60].

### Synthesis of pyrazolo-1,2-benzothiazine derivative **5**

A solution of compound **4** (269 mg, 1 mmol) in ethanol (10 mL) was refluxed with excess of hydrazine monohydrate for 24 hours. After the completion of reaction, unreacted hydrazine was removed under vacuum. Cold acidified water was poured into the residue. Yellow-colored precipitates of product **5** were formed which were separated via filtration followed by washing with excess of water. After drying, recrystallization was done with ethanol. Mp 265–266 °C; yield: 221 mg (88%).

### Synthesis of pyrazolo-1,2-benzothiazine-*N*-aryl/benzyl/cyclohexylacetamides **7a–h**

Dried acetonitrile (10 mL) was used to prepare a solution of compound **5** (251 mg, 1.0 mmol) followed by addition of anhydrous K_2_CO_3_ (173 mg, 1.25 mmol) under continuous stirring and heating. After 30 minutes, the solution of the respective 2-chloro-*N*-aryl/benzyl/cyclohexylacetamide **6a**–**h** (1 mmol) in dry acetonitrile was added dropwise. Then, the reaction mixture was continued to reflux for 24 h or until the termination of the reaction. Reaction progress was monitored after intervals using TLC. On completion, filtration of the reaction mixture was performed followed by concentration under vacuum. It was then diluted with cold water and acidified with 5% cold HCl. The solution was allowed to stand for 15 minutes to complete precipitation. Precipitates were collected via filtration and washed with excess distilled water. The dried product was then recrystallized from absolute ethanol.

### Synthesis of pyrazolo-1,2-benzothiazine-*N*-aryl/benzyl/cyclohexylacetamides **7i–n**

Dried acetonitrile (10 mL) was used to prepare a solution of compound **5** (1.0 mmol, 251 mg) followed by the addition of anhydrous K_2_CO_3_ (297 mg, 2.15 mmol) under continuous stirring and heating. After 30 minutes, the solution of respective 2-chloro-*N*-aryl/benzyl/cyclohexylacetamides **7i**–**n** (2 mmol) in dry acetonitrile was added dropwise. The mixture was heated to reflux for 40 h or until the termination of the reaction. Reaction progress was monitored after intervals using TLC. On completion, filtration of the reaction mixture was performed followed by concentration under vacuum. It was then diluted with cold water and acidified with 5% cold HCl. The solution was allowed to stand for 15 minutes to complete precipitation. Precipitates were collected via filtration and washed with excess distilled water. The dried product was then recrystallized from absolute ethanol.

### Characterization data

Solutions of all synthesized compounds **7a**–**h** were prepared in DMSO-*d*_6_ and scanned for their ^1^H NMR spectra at 300 MHz and ^13^C NMR spectra at 75 MHz. The characterization data for compounds **7a**–**h** is given in Supporting Information File 1.

### Antimicrobial assays

The antimicrobial evaluation of the targeted compounds was carried out against five different pathogens including *Staphylococcus aureus* (susceptible, ATCC 25923; methicillin-resistant, ATCC BAA-41; multidrug-resistant, ATCC BAA-44), *Escherichia coli* ATCC 8739, and *Candida albicans* ATCC 90027. Reference antibiotics and final concentrations used as positive controls in these experiments were kanamycin (susceptible *S. aureus*, 50 μg), vancomycin (methicillin and multidrug-resistant *S. aureus*, 25 μg), ampicillin (*E. coli*, 50 μg), and amphotericin B (*C. albicans*, 25 μg). The final concentration of the tested compounds was 125 μg/mL for microbroth single-dose assays. Dimethyl sulfoxide was used at a final concentration of 0.1% as negative control. Microbroth dilution assays [52] for the most potent bioactive compounds **7b** and **7h** were performed using nine serial dilutions of the tested compounds with final concentrations of 256, 128, 64, 32, 16, 8, 4, 2, and 1 μg/mL. To assess turbidity of wells, absorbance was measured at 620 nm by using a Bioteck Synergy reader (96-well plate) and hence microbial growth rates were obtained. American type culture collection (ATCC, Manassas, VA, USA) has provided all microbial strains used in above experiments.

### Cytotoxic assays

Single-dose MTT assays were performed for compounds **7a**–**n** with the human colon cancer mammalian cell line (HCT-116, ATCC CCL-247) for evaluating the compounds’ inhibitory effects on cell viability [53–56]. Stock solutions of compounds were prepared in DMSO and cancer cells were treated with 10 μM doses of each compound and mensacarcin (10 μM) as a positive control [56]. MTT in 1X PBS was added for a final concentration of 0.5 mg/mL 48 hours post compound addition to each well. Post incubation at 37 °C was carried out for 2 h. After the removal of the growth media, purple formazan metabolic product was dissolved in DMSO. A Biotek Synergy reader (96-well plate) was used to take absorbance of all samples at 550 nm. Cell viability was calculated as the ability of the metabolically active cells to reduce tetrazolium salt MTT [3-(4,5-dimethylthiazolyl-2)-2,5-diphenyltetrazolium bromide]. Control metabolic activity shown by cells treated with 0.1% DMSO was set to 100% cell growth.

### CA inhibition assays

The inhibition potential of the synthesized compound **7a**–**h** against different hCAs (I, II, IX, XII) was analyzed by adopting a stopped-flow CO_2_ hydration method described by Martínez-Montiel and colleagues in 2023 [57]. Analyses were carried out in-house using recombinant enzymes as described. Enzyme concentrations (5–12 nM) used in these assays were the same as previously reported [57,61].

## Supporting Information

File 1 Experimental procedures, spectra (NMR, HRMS) and graphs of antimicrobial and cytotoxic assays.

## Data Availability

All data that supports the findings of this study is available in the published article and/or the supporting information of this article.
