# Supplementary material for: Synthesis, characterization, antimicrobial, cytotoxic and carbonic anhydrase inhibition activities of multifunctional pyrazolo-1,2-benzothiazine acetamides
Source: Beilstein J Org Chem. 2025 Feb 12;21:348–57. doi: 10.3762/bjoc.21.25 (PMC11833175; doi:10.3762/bjoc.21.25)
Supplement: File 1 — Experimental procedures, spectra (NMR, HRMS) and graphs of antimicrobial and cytotoxic assays. [file Beilstein_J_Org_Chem-21-348-s001.pdf]

BEILSTEIN JOURNAL OF ORGANIC CHEMISTRY

Supporting Information

for

Synthesis, characterization, antimicrobial, cytotoxic and carbonic anhydrase inhibition activities of multifunctional pyrazolo-1,2-benzothiazine acetamides

Ayesha Saeed, Shahana Ehsan, Muhammad Zia-ur-Rehman, Erin M. Marshall, Sandra Loesgen, Abdus Saleem, Simone Giovannuzzi and Claudiu T. Supuran

Beilstein J. Org. Chem. **2025,** *21,* 348–357. doi:10.3762/bjoc.21.25

Experimental procedures, spectra (NMR, HRMS) and graphs of antimicrobial and cytotoxic assays

License and Terms: This is a supporting information file under the terms of the Creative Commons Attribution License (https://creativecommons.org/licenses/by/4.0). Please note that the reuse, redistribution and reproduction in particular requires that the author(s) and source are credited and that individual graphics may be subject to special legal provisions.
The license is subject to the *Beilstein Journal of Organic Chemistry* terms and conditions: (https://www.beilstein-journals.org/bjoc/terms)

Table of contents

Sr. #	Contents	Page #
**1**	**Experimental procedures**	**S3**
1.1	Synthesis of methyl (1,1-dioxido-3-oxo-1,2-benzisothiazol-2(3*H*)-yl)acetate (**2**)	S3
1.2	Synthesis of methyl 4-hydroxy-2*H*-1,2-benzothiazine-3-carboxylate 1,1-dioxide (**3**)	S3
1.3	Synthesis of methyl 4-hydroxy-2-methyl-*2H*-benzo[*e*][1,2]thiazine-3-carboxylate 1,1-dioxide (**4**)	S3
1.4	Synthesis of 2-chloro-*N*-aryl/benzyl/cyclohexylacetamides **6a**–**n**	S4
**2**	**Spectra of pyrazolo-1,2-benzothiazine acetamides 7a–n**	**S4**
Figure S1	^{1}H NMR spectrum of compound **7a** in DMSO-d_6	S4
Figure S2	^{13}C NMR spectrum of compound **7a** in DMSO-d_6	S5
Figure S3	HRMS of compound **7a**	S5
Figure S4	^{1}H NMR spectrum of compound **7b** in DMSO-d_6	S6
Figure S5	^{13}C NMR spectrum of compound **7b** in DMSO-d_6	S6
Figure S6	HRMS of compound **7b**	S7
Figure S7	^{1}H NMR spectrum of compound **7c** in DMSO-d_6	S7
Figure S8	^{13}C NMR spectrum of compound **7c** in DMSO-d_6	S8
Figure S9	HRMS of compound **7c**	S8
Figure S10	^{1}H NMR spectrum of compound **7d** in DMSO-d_6	S9
Figure S11	^{13}C NMR spectrum of compound **7d** in DMSO-d_6	S9
Figure S12	HRMS of compound **7d**	S10
Figure S13	^{1}H NMR spectrum of compound **7e** in DMSO-d_6	S10
Figure S14	^{13}C NMR spectrum of compound **7e** in DMSO-d_6	S11
Figure S15	HRMS of compound **7e**	S11
Figure S16	^{1}H NMR spectrum of compound **7f** in DMSO-d_6	S12
Figure S17	^{13}C NMR spectrum of compound **7f** in DMSO-d_6	S12
Figure S18	HRMS of compound **7f**	S13
Figure S19	^{1}H NMR spectrum of compound **7g** in DMSO-d_6	S13
Figure S20	^{13}C NMR spectrum of compound **7g** in DMSO-d_6	S14
Figure S21	HRMS of compound **7g**	S14
Figure S22	^{1}H NMR spectrum of compound **7h** in DMSO-d_6	S15
Figure S23	^{13}C NMR spectrum of compound **7h** in DMSO-d_6	S15
Figure S24	HRMS of compound **7h**	S16

Figure S25	^{1}H NMR spectrum of compound **7i** in DMSO-d_6	S16
Figure S26	^{13}C NMR spectrum of compound **7i** in DMSO-d_6	S17
Figure S27	HRMS of compound **7i**	S17
Figure S28	^{1}H NMR spectrum of compound **7j** in DMSO-d_6	S18
Figure S29	^{13}C NMR spectrum of compound **7j** in DMSO-d_6	S18
Figure S30	HRMS of compound **7j**	S19
Figure S31	^{1}H NMR spectrum of compound **7k** in DMSO-d_6	S19
Figure S32	^{13}C NMR spectrum of compound **7k** in DMSO-d_6	S20
Figure S33	HRMS of compound **7k**	S20
Figure S34	^{1}H NMR spectrum of compound **7l** in DMSO-d_6	S21
Figure S35	^{13}C NMR spectrum of compound **7l** in DMSO-d_6	S21
Figure S36	HRMS of compound **7l**	S22
Figure S37	^{1}H NMR spectrum of compound **7m** in DMSO-d_6	S22
Figure S38	^{13}C NMR spectrum of compound **7m** in DMSO-d_6	S23
Figure S39	HRMS of compound **7m**	S23
Figure S40	^{1}H NMR spectrum of compound **7n** in DMSO-d_6	S24
Figure S41	^{13}C NMR spectrum of compound **7n** in DMSO-d_6	S24
Figure S42	HRMS of compound **7n**	S25
Figure S43	Graph showing results of antimicrobial single-dose microbroth assays of compounds **7a**–**n**	S26
Table S1	Results of single-dose antimicrobial and cytotoxic assays for compounds **7a**–**n**	S27
Figure S44	Graph showing cytotoxicity of compounds **7a–n** against human colon carcinoma cell line (HCT-116)	S28
**3**	**Characterization data of compounds 7a–n**	**S27**
	**References**	**S34**

1. Experimental procedures

1.1 Synthesis of methyl 2-(1,1-dioxido-3-oxo-1,2-benzisothiazol-2(3*H*)-yl)acetate (2)

Saccharine sodium (5.125 g, 25 mmol) was dissolved in DMF (30 mL) and methyl chloroacetate (2.712 g, 25 mmol) was added after the complete dissolution of saccharine sodium. The reaction mixture was stirred at 90–110 °C for 3–4 hours. After the completion of the reaction, the reaction mixture was allowed to cool to room temperature. Now the flask contents were poured into ice-cold water. White precipitates of methyl 2-(1,1-dioxido-3-oxobenzo[*d*]isothiazol-2(*3H*)-yl)acetate (**2**) were formed which were filtered, washed with excess of water and then recrystallized from ethanol [1,2]. M.P: 116-117ºC, Yield: 5.487 g (86%).

1.2 Synthesis of methyl 4-hydroxy-2*H*-benzo[*e*][1,2]thiazine-3-carboxylate 1,1-dioxide (3)

Fresh sodium methoxide (1.350 g, 25 mmol) was prepared using sodium metal and dry methanol (30 mL) as solvent. A solution of compound **2** (2.550 g, 10 mmol) in dry DMSO was prepared separately. Both of these solutions were prepared under moisture-free conditions. Then these two solutions were mixed at room temperature and swirled well until a dark orange red color was obtained. Then, the contents of the reaction mixture were poured over the mixture of ice and concentrated hydrochloric acid having pH 3. Keep on stirring while pouring the reaction mixture. White colored precipitates of methyl 4-hydroxy-*2H*-benzo[*e*][1,2]thiazine-3-carboxylate 1,1-dioxide (**3**) were formed, which were then filtered, washed with excess water, dried and recrystallized using ethanol [1,2]. M.P: 171-172ºC, Yield: 1.531 g (60%).

1.3 Synthesis of methyl 4-hydroxy-2-methyl-*2H*-benzo[*e*][1,2]thiazine-3-carboxylate 1,1-dioxide (4)

Compound **3** (5.100 g, 20 mmoles) was dissolved in acetone (20 mL) and sodium hydroxide (2 N) was added slowly while stirring at room temperature to set the pH at 10–11. The reaction mixture was stirred for 5 minutes. Then, dimethyl sulfate (2.520 g, 20 mmol) was added drop-wise. This reaction mixture was further stirred for 40 minutes. After the completion of the reaction, it was acidified with dilute hydrochloric acid until the appearance of white precipitates of methyl 4-hydroxy-2-methyl-*2H*-benzo[*e*][1,2]thiazine-3-carboxylate 1,1-dioxide (**4**). These were filtered, washed with water, dried and recrystallized with ethanol [3]. M.P 161-162ºC, Yield: 3.822 g (71%).

1.4 Synthesis of 2-chloro-*N*-aryl/benzyl/cyclohexylacetamides 6a–n

Aryl/benzyl/cyclohexylamines (5 mmol) were dissolved in DCM and Na_2CO_3 solution (7%) was added dropwise to set the pH at 9.0. This reaction mixture was stirred for 30 minutes at room temperature. Then, chloroacetyl chloride (847 mg, 7.5 mmol) in 2 mL DCM was added drop-wise in about 10–15 min with vigorous stirring. Stirring was continued until the precipitation of solid 2-chloro-*N*-aryl/benzyl/cyclohexylacetamides **6a**–**n**. After the completion of the reaction, precipitates were filtered, washed with chilled distilled water, and dried to produce the desired alkylating agents [4].

2. Spectra of pyrazolo-1,2-benzothiazine acetamides 7a–n

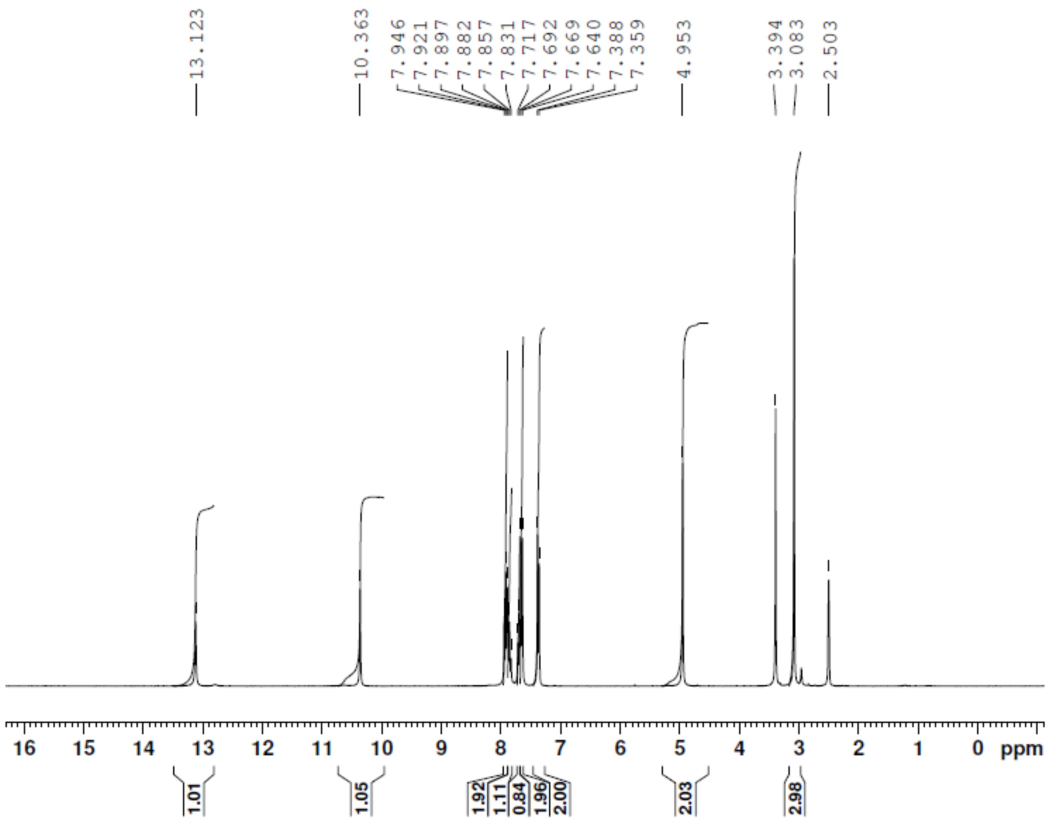

Figure S1. ^{1}H NMR of compound **7a** in DMSO-d_6 at 300 MHz.

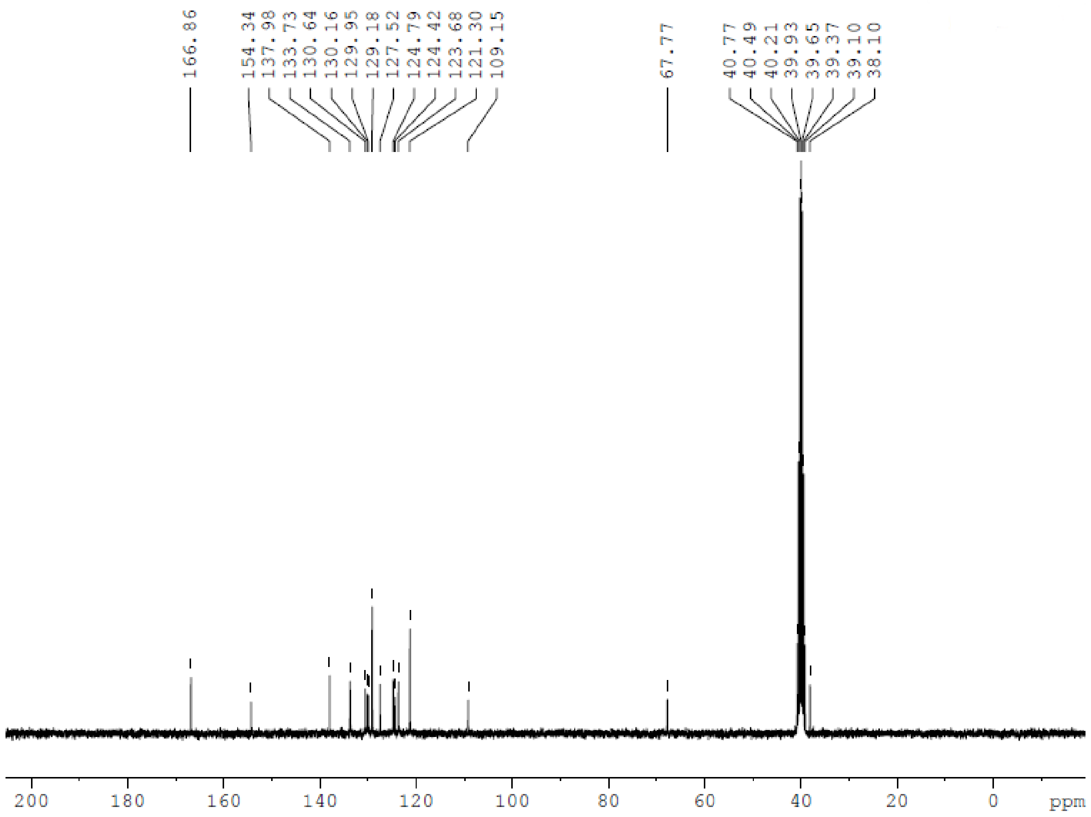

Figure S2. ^{13}C NMR of compound **7a** in DMSO-d_6 at 75 MHz.

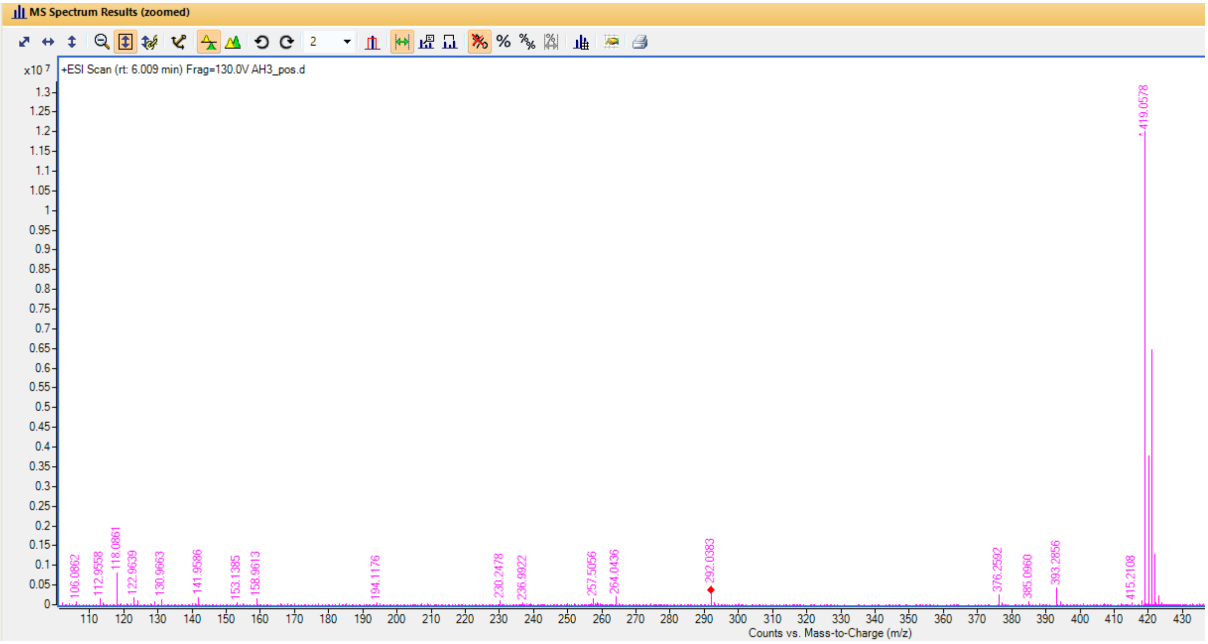

Figure S3. HRMS of compound **7a.**

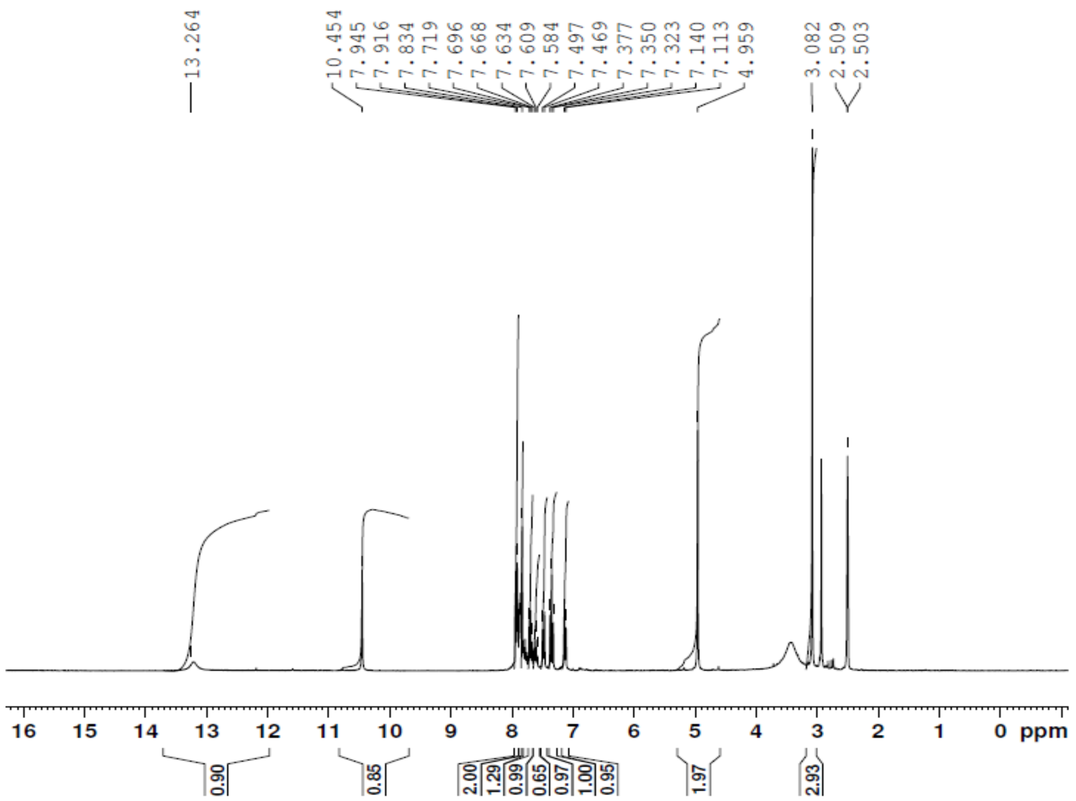

Figure S4. ^{1}H NMR of compound **7b** in DMSO-d_6 at 300 MHz.

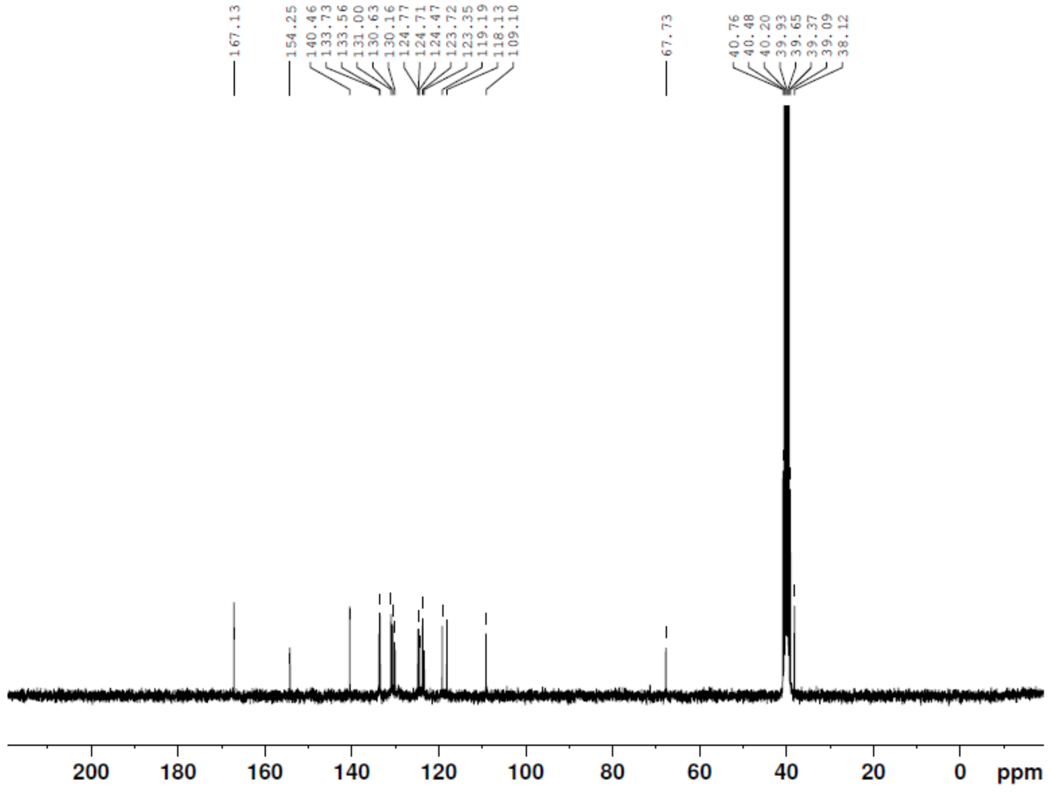

Figure S5. ^{13}C NMR of compound **7b** in DMSO-d_6 at 75 MHz.

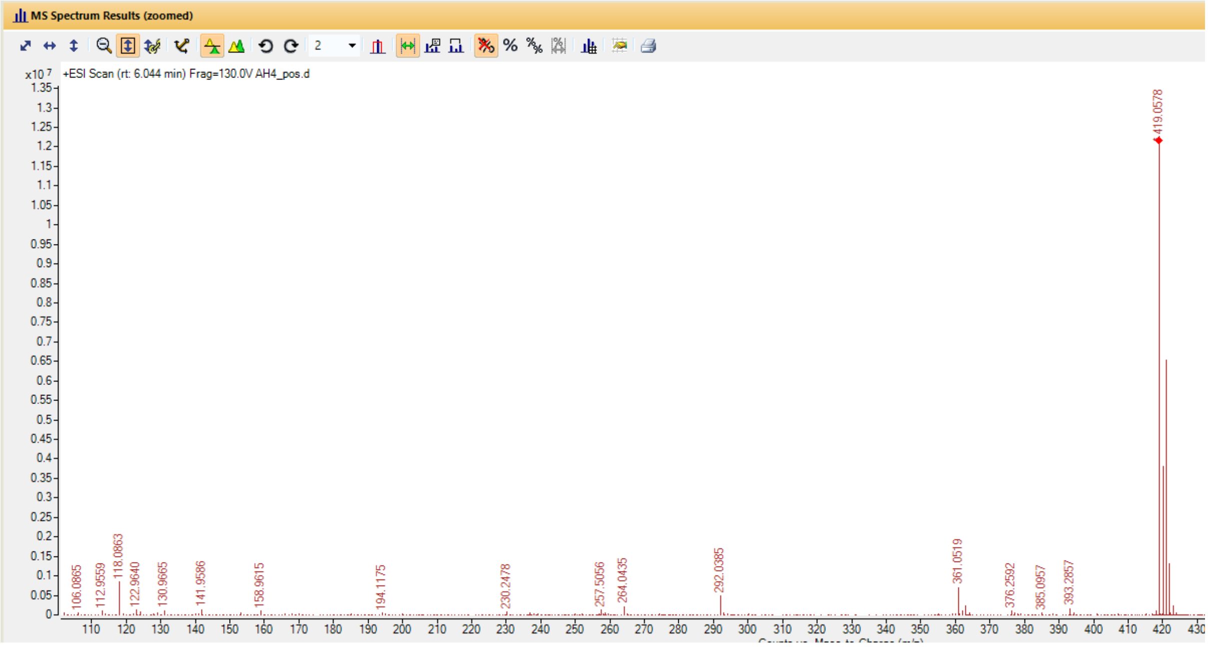

Figure S6. HRMS of compound **7b**.

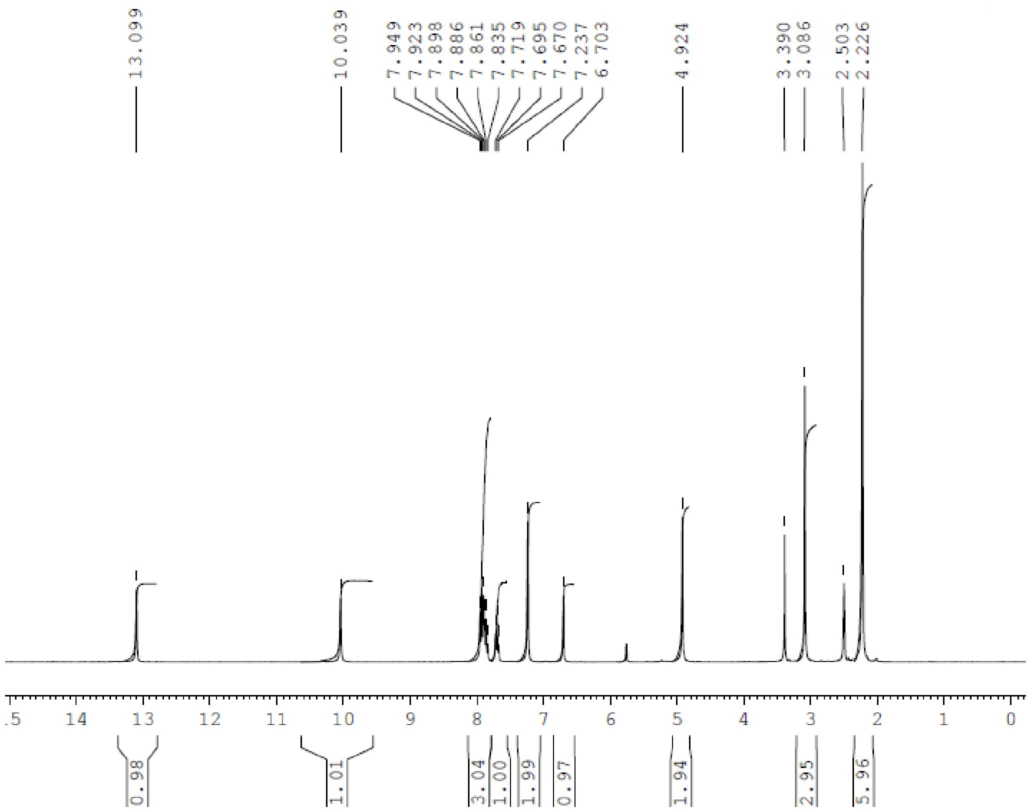

Figure S7. ^{1}H NMR of compound **7c** in DMSO-d_6 at 300 MHz.

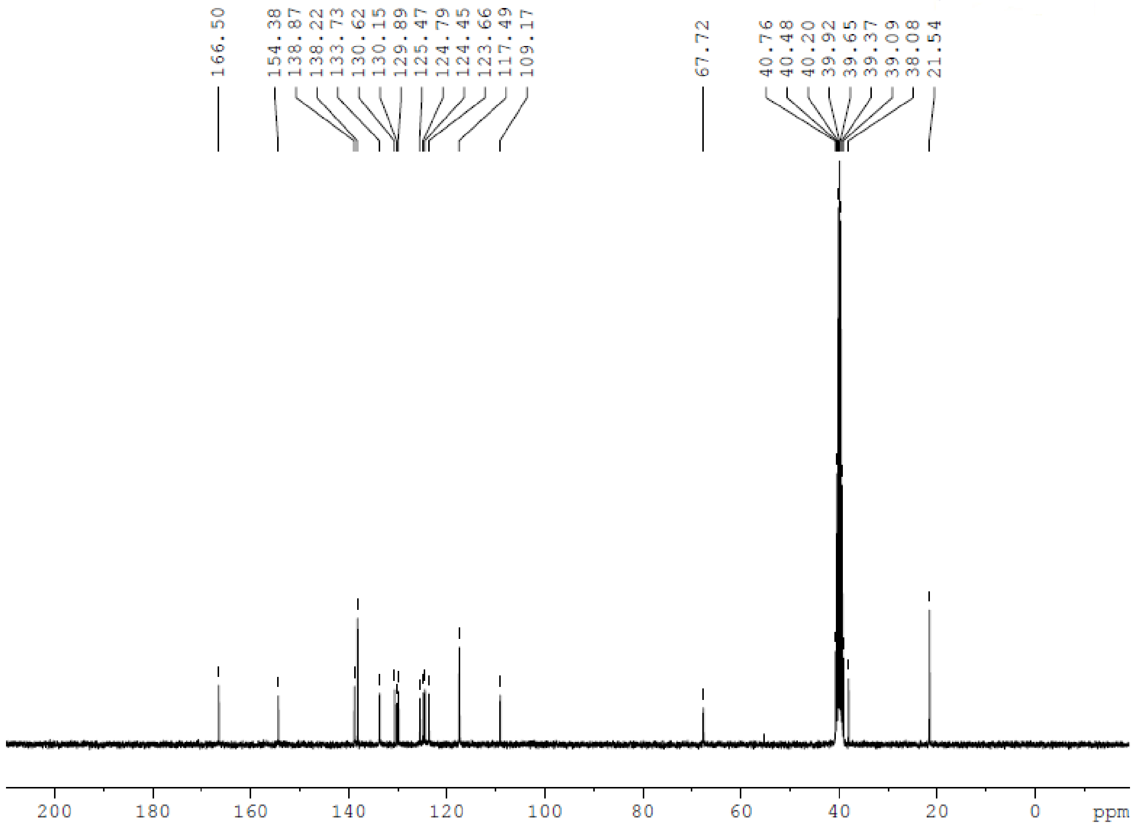

Figure S8. ^{13}C NMR of compound **7c** in DMSO-d$_6$ at 75 MHz.

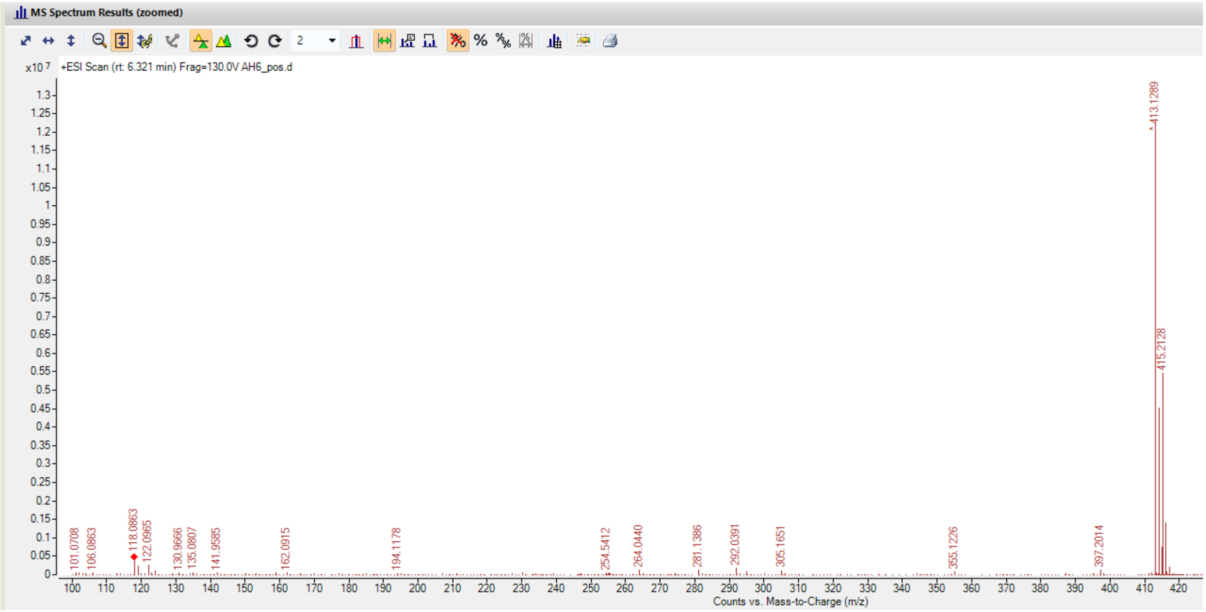

Figure S9. HRMS of compound **7c**.

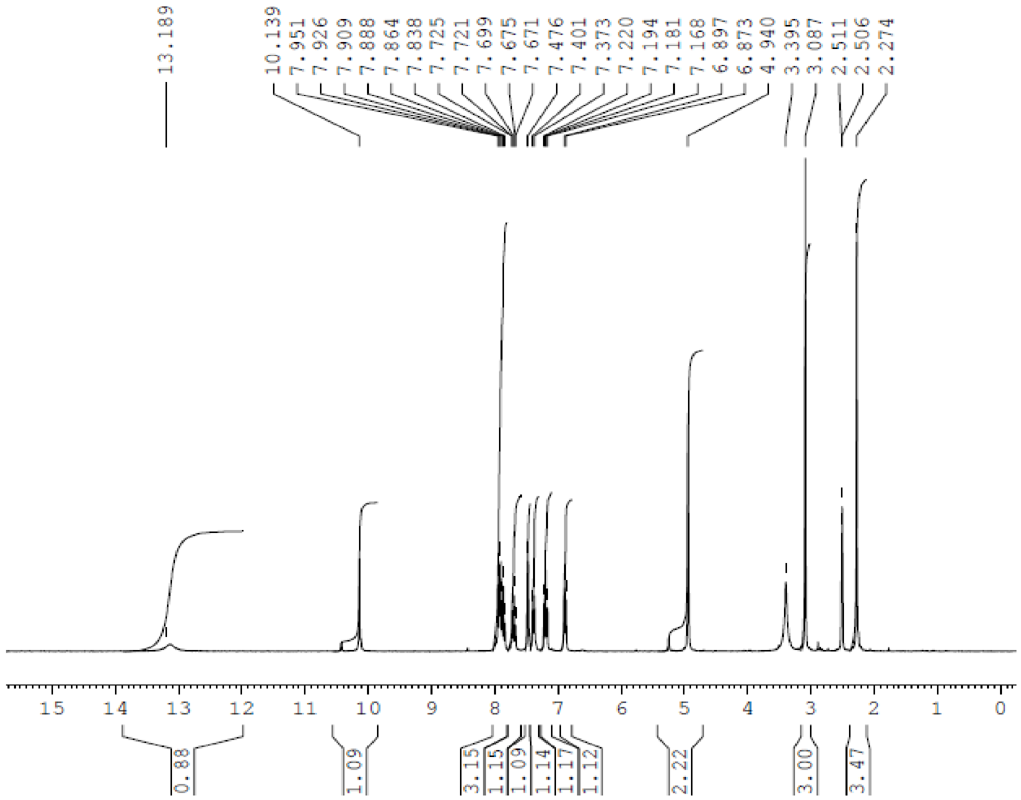

Figure S10. ^{1}H NMR of compound **7d** in DMSO-d_6 at 300 MHz.

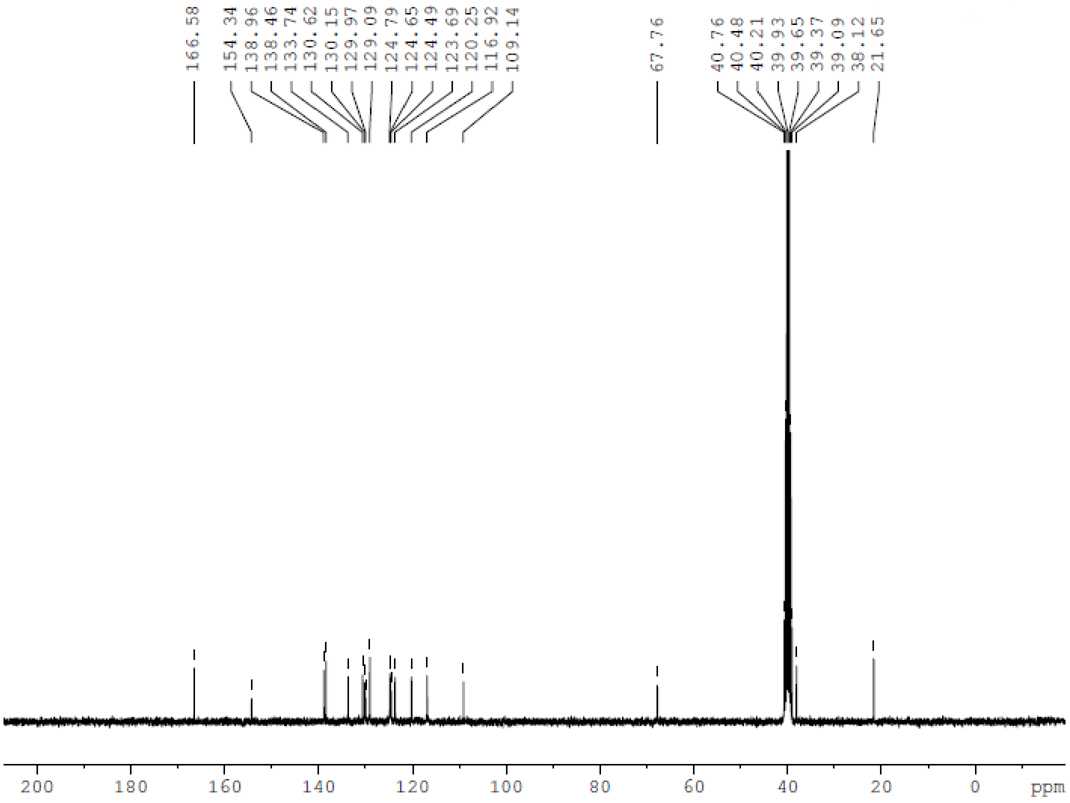

Figure S11. ^{13}C NMR of compound **7d** in DMSO-d_6 at 75 MHz.

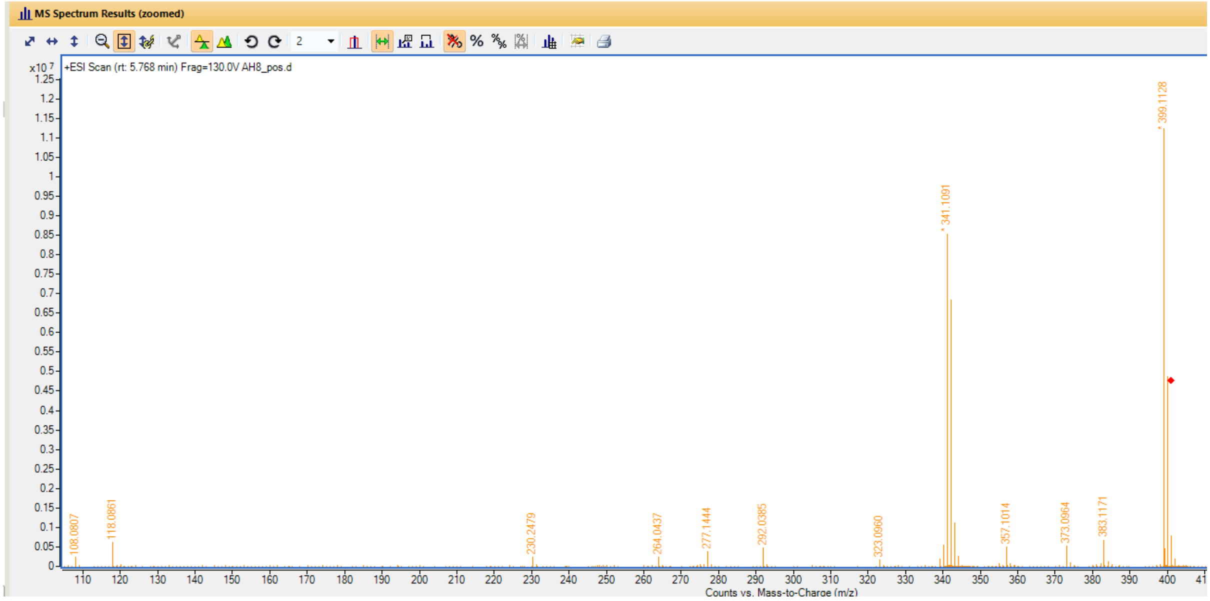

Figure S12. HRMS of compound **7d**.

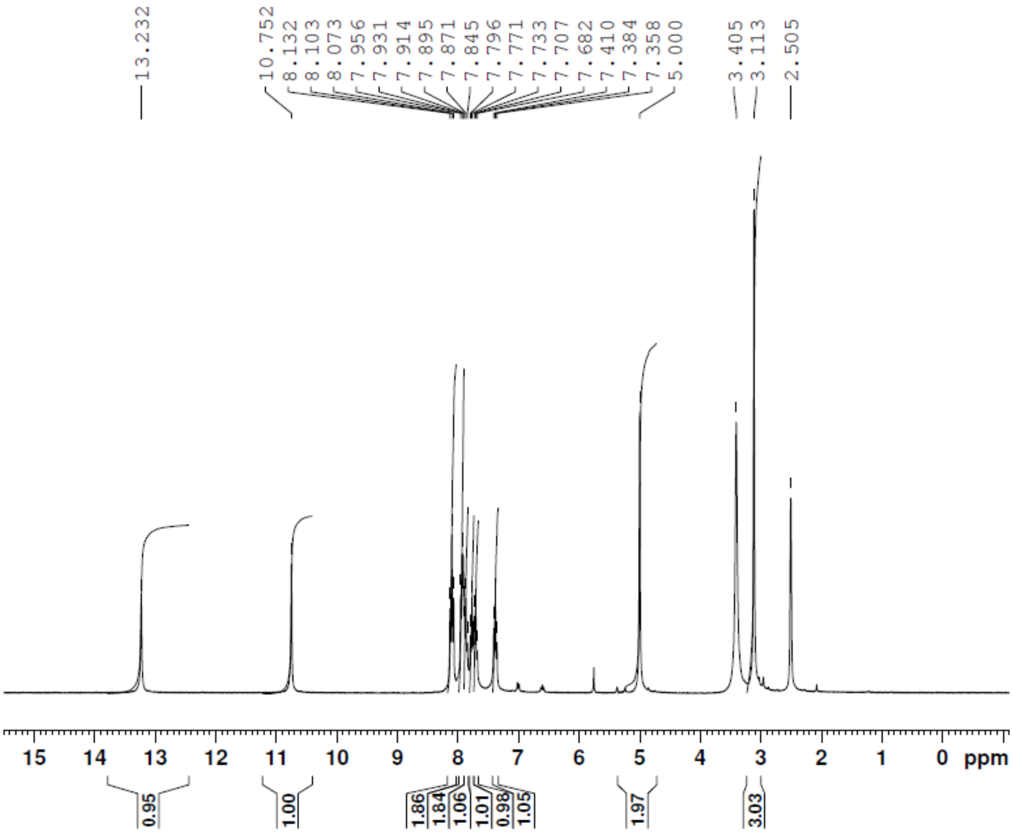

Figure S13. ^{1}H NMR of compound **7e** in DMSO-d_6 at 300 MHz.

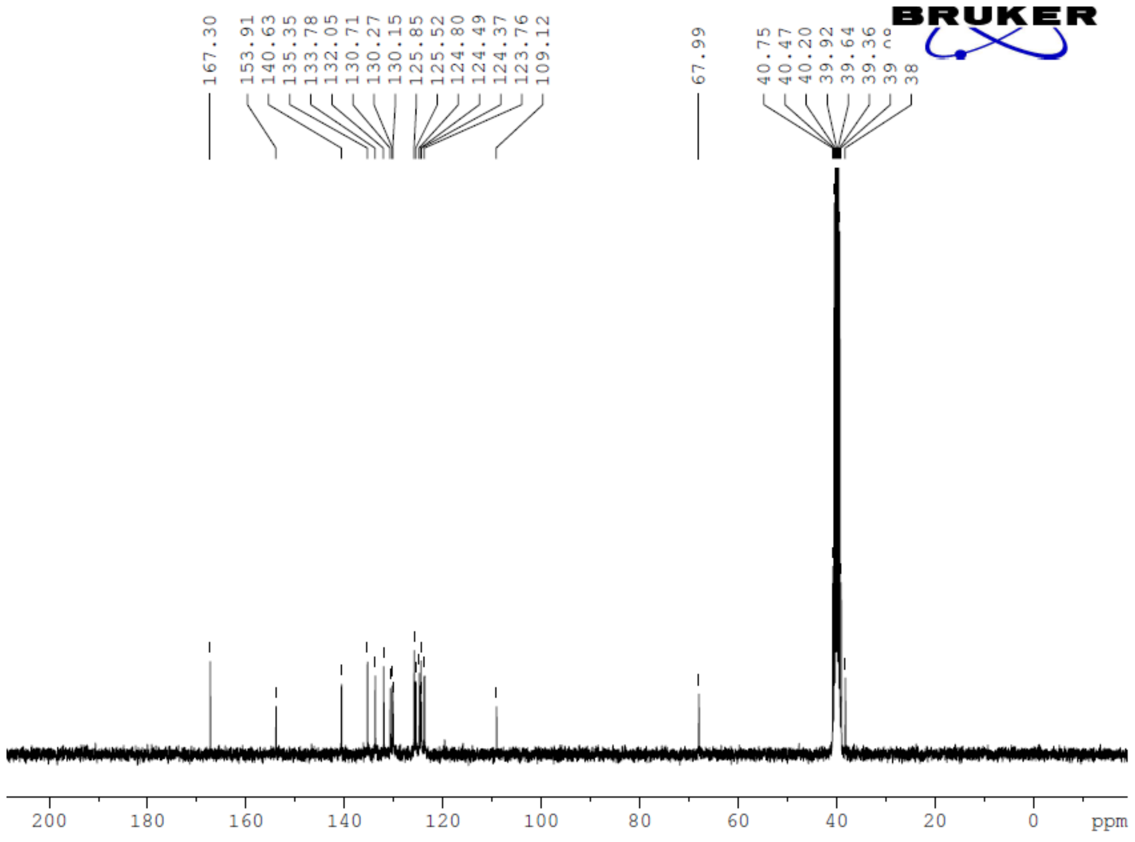

Figure S14. ^{13}C NMR of compound **7e** in DMSO-d_6 at 75 MHz.

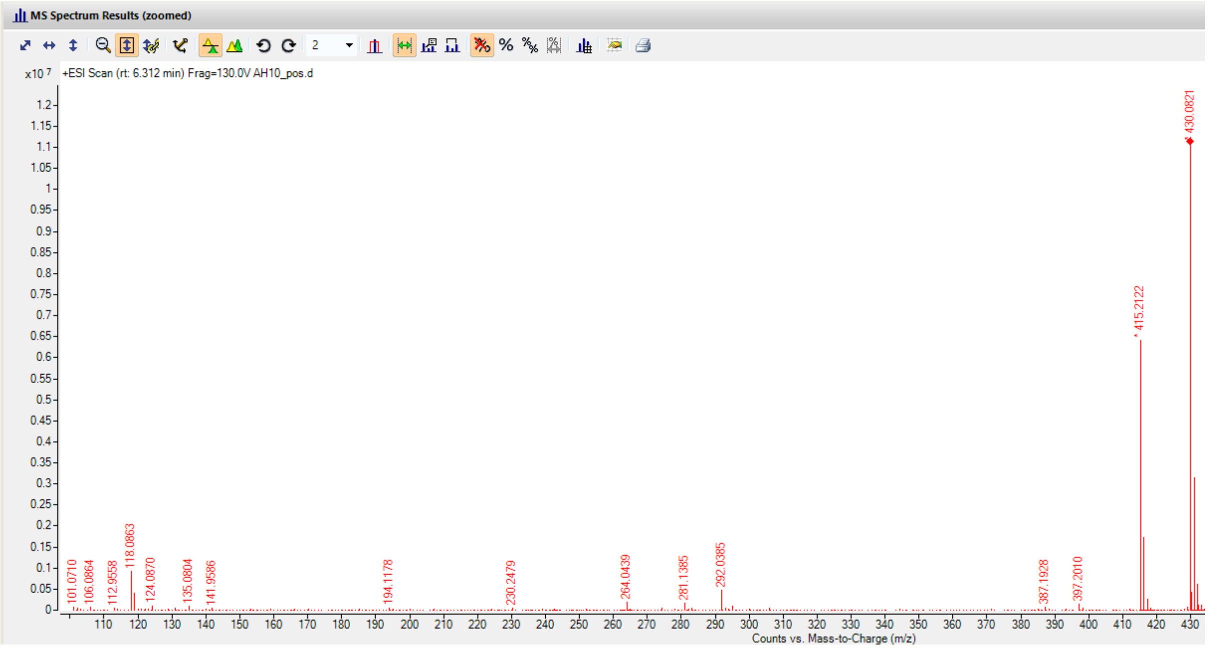

Figure S15. HRMS of compound **7e**.

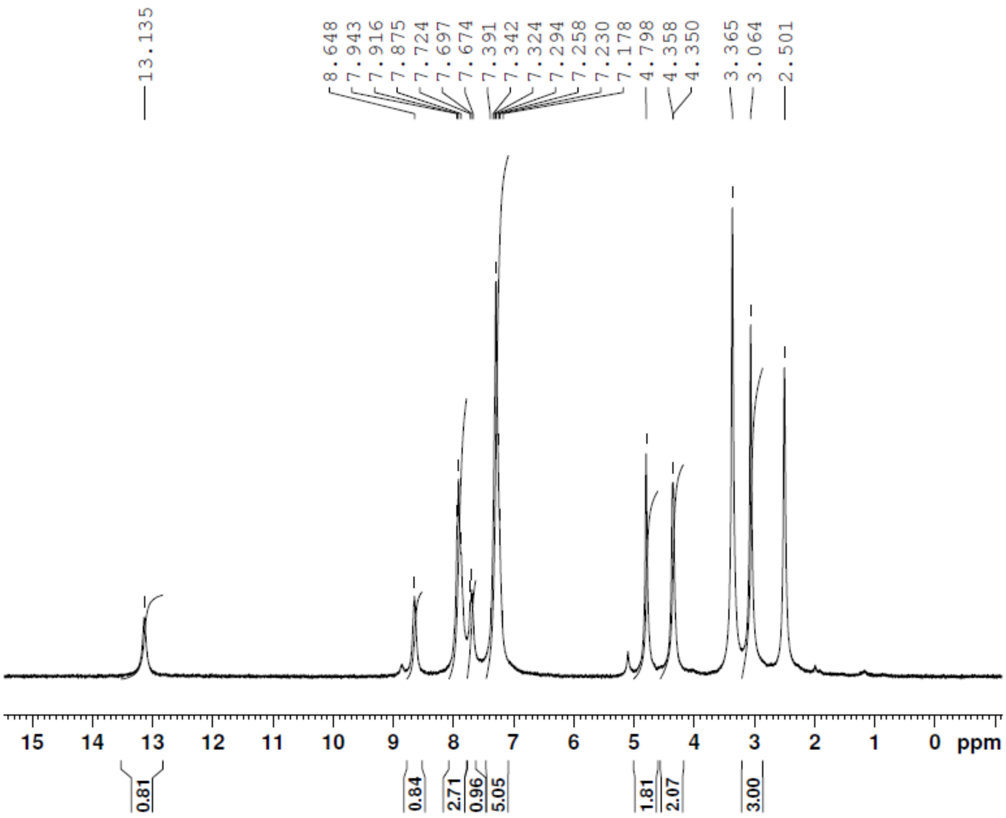

Figure S16. ^{1}H NMR of compound **7f** in DMSO-d_6 at 300 MHz.

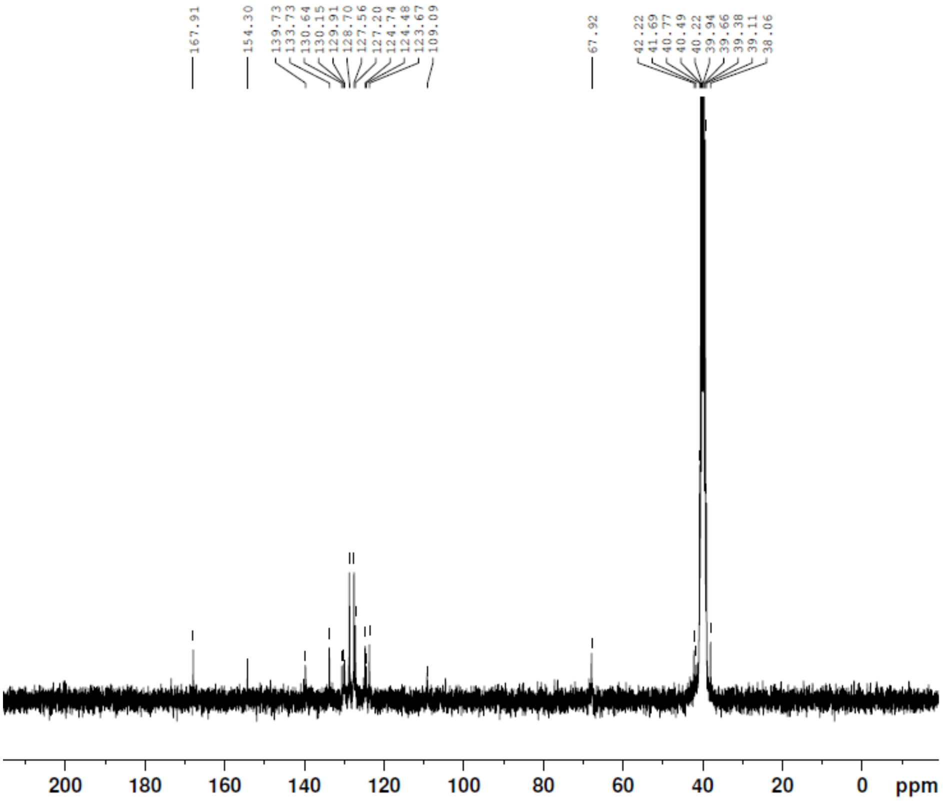

Figure S17. ^{13}C NMR of compound **7f** in DMSO-d_6 at 75 MHz.

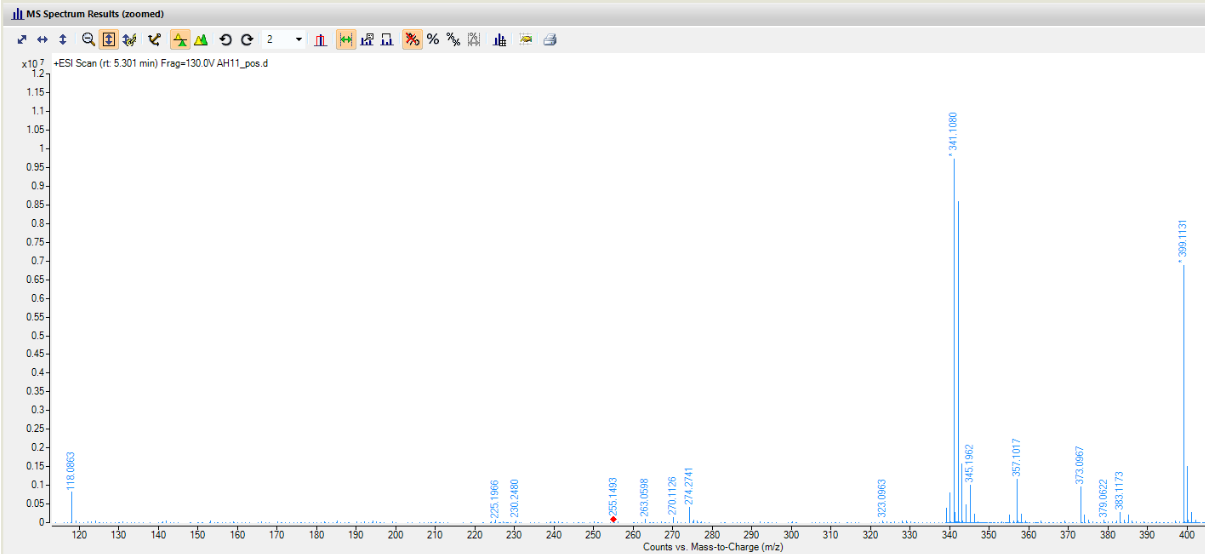

Figure S18. HRMS of compound **7f**.

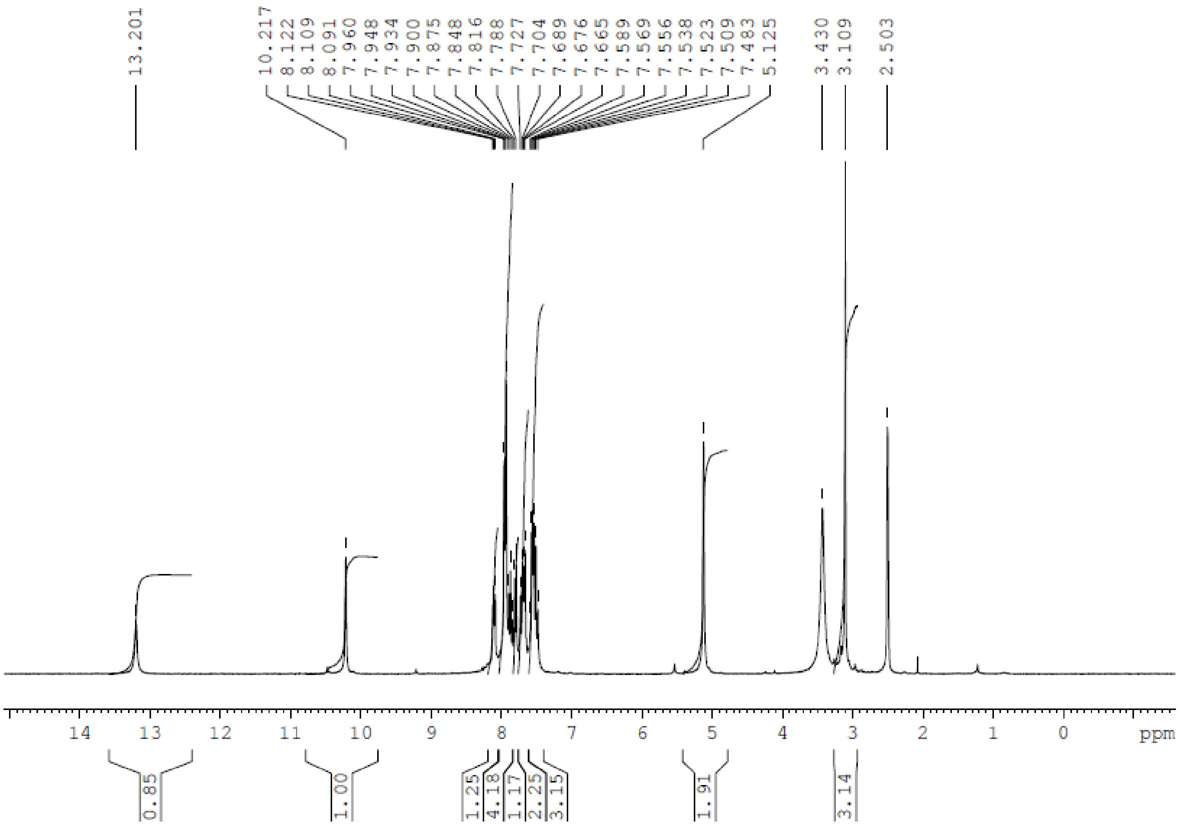

Figure S19. ^{1}H NMR of compound **7g** in DMSO-d6 at 300 MHz.

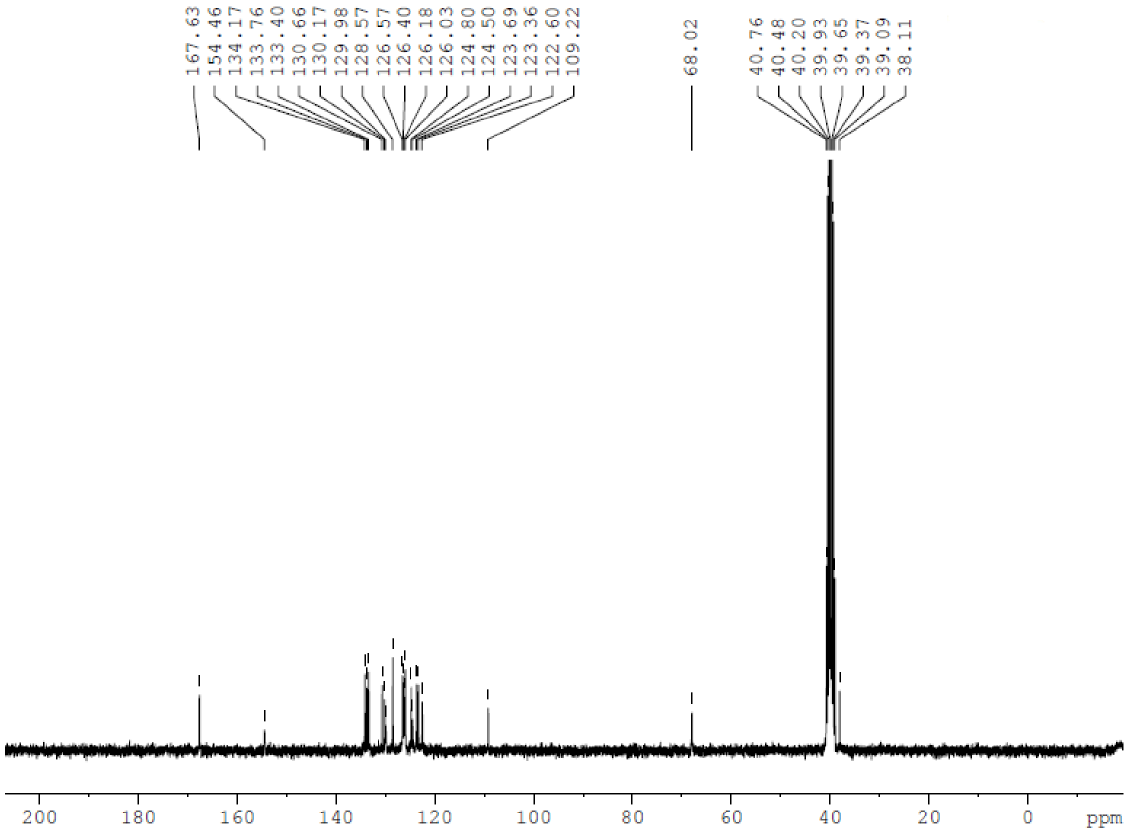

Figure S20. ^{13}C NMR of compound **7g** in DMSO-d_6 at 75 MHz.

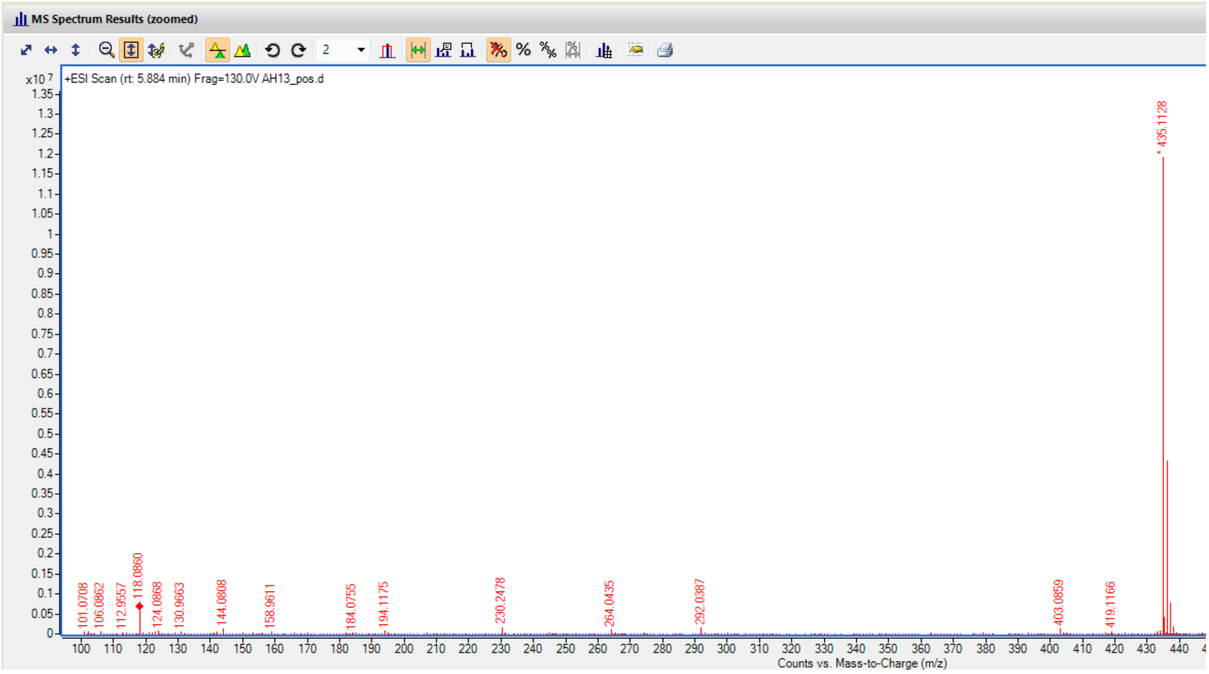

Figure S21. HRMS of compound **7g**.

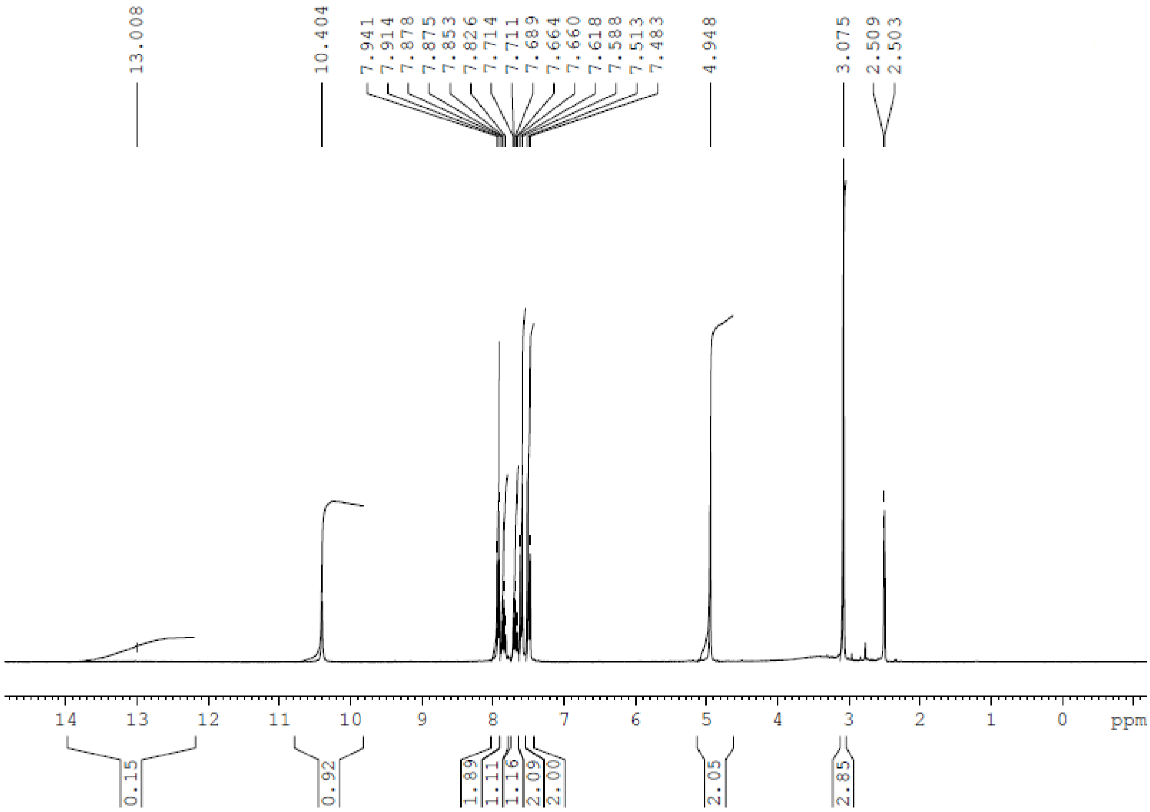

Figure S22. ^{1}H NMR of compound **7h** in DMSO-d_6 at 300 MHz.

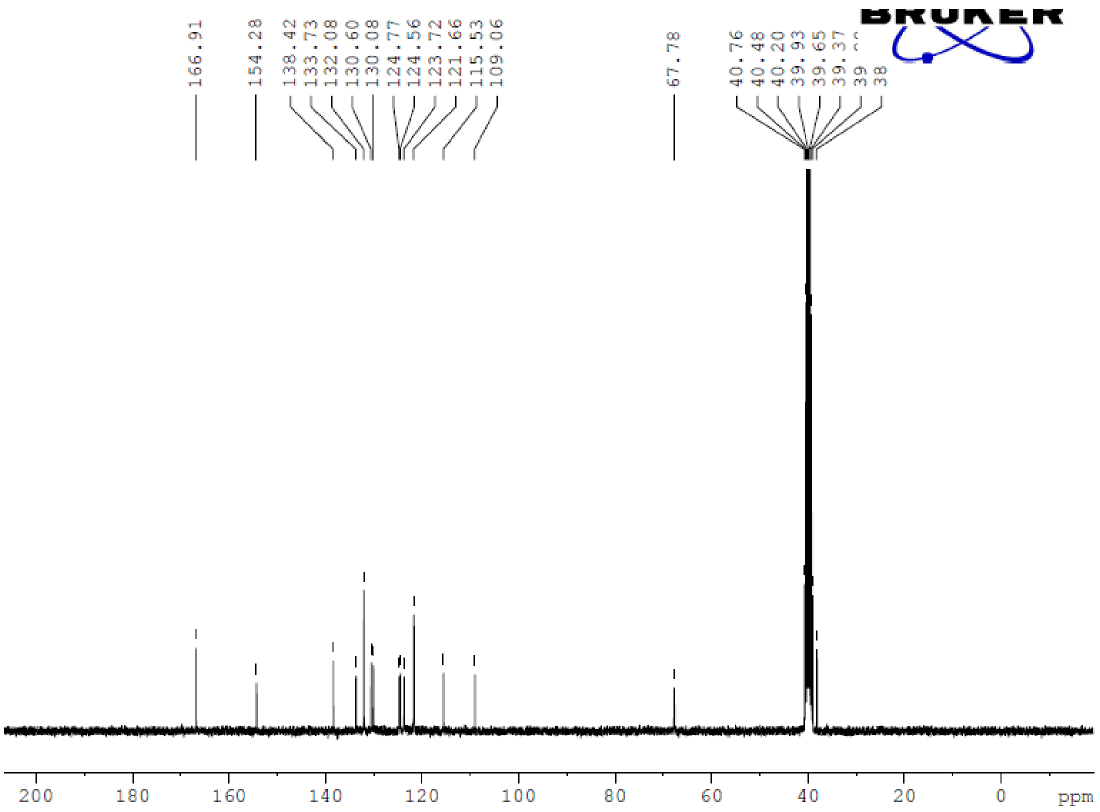

Figure S23. ^{13}C NMR of compound **7h** in DMSO-d_6 at 75 MHz.

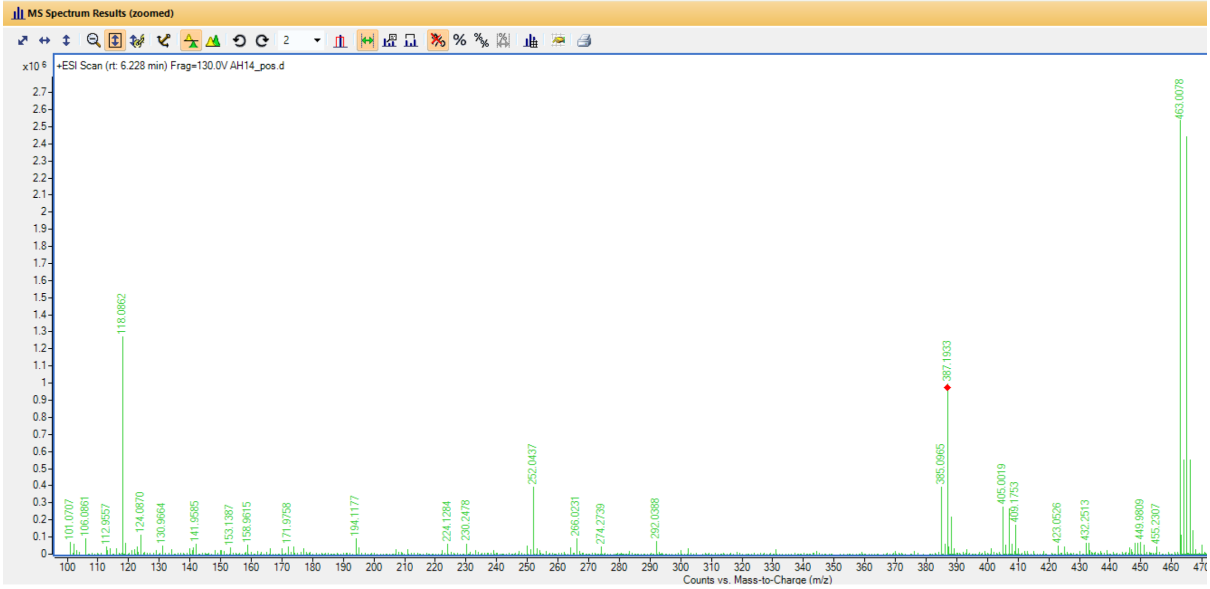

Figure S24. HRMS of compound **7h**.

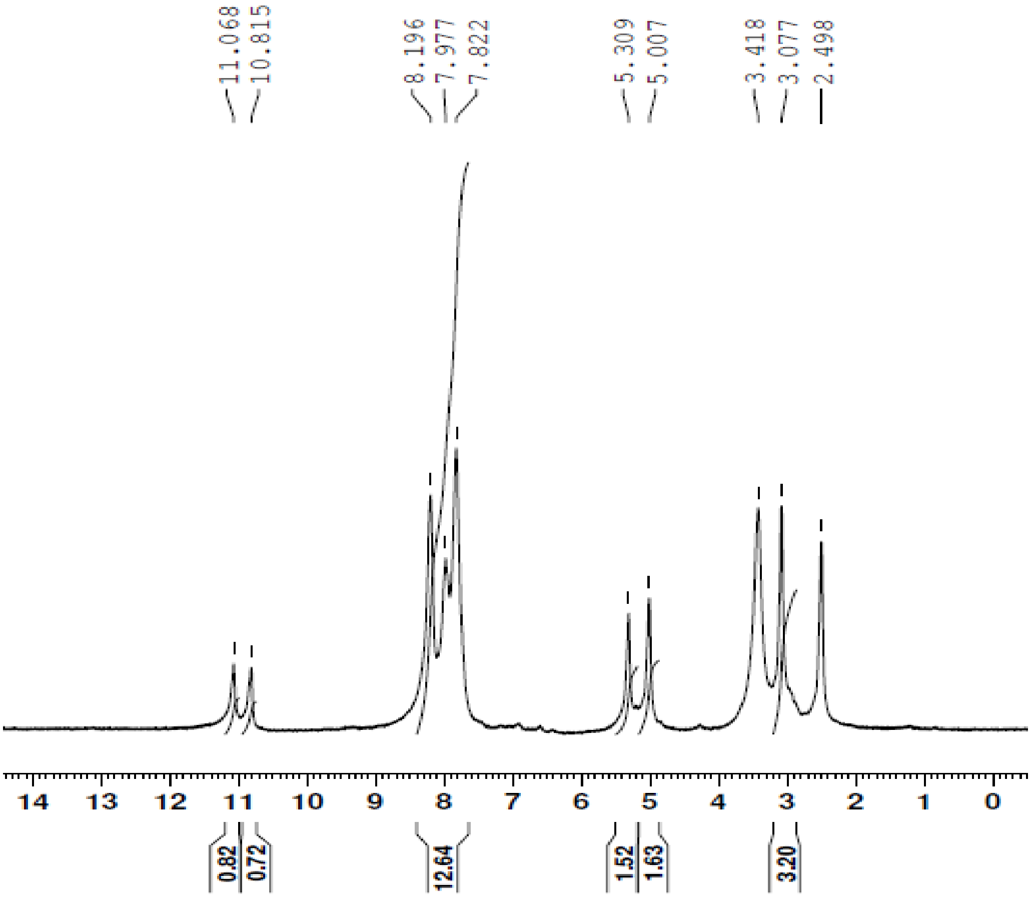

Figure S25. ^{1}H NMR of compound **7i** in DMSO-d_6 at 300 MHz.

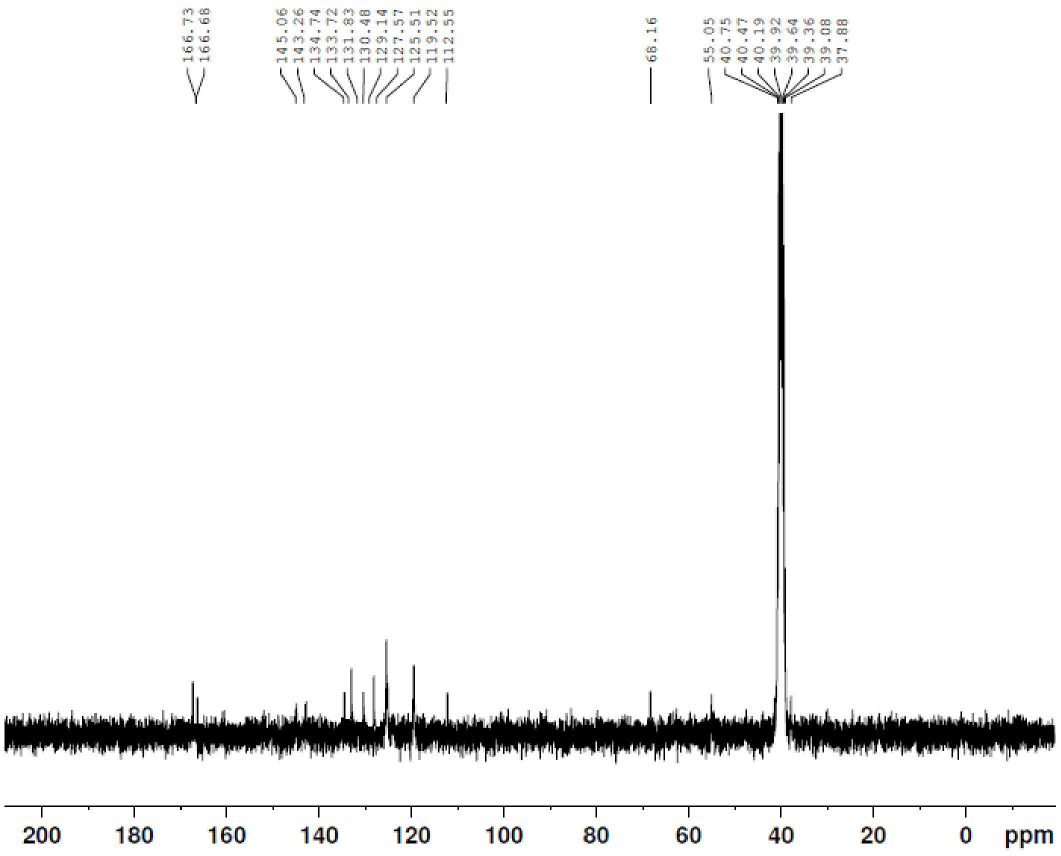

Figure S26. ^{13}C NMR of compound **7i** in DMSO-d_6 at 75 MHz.

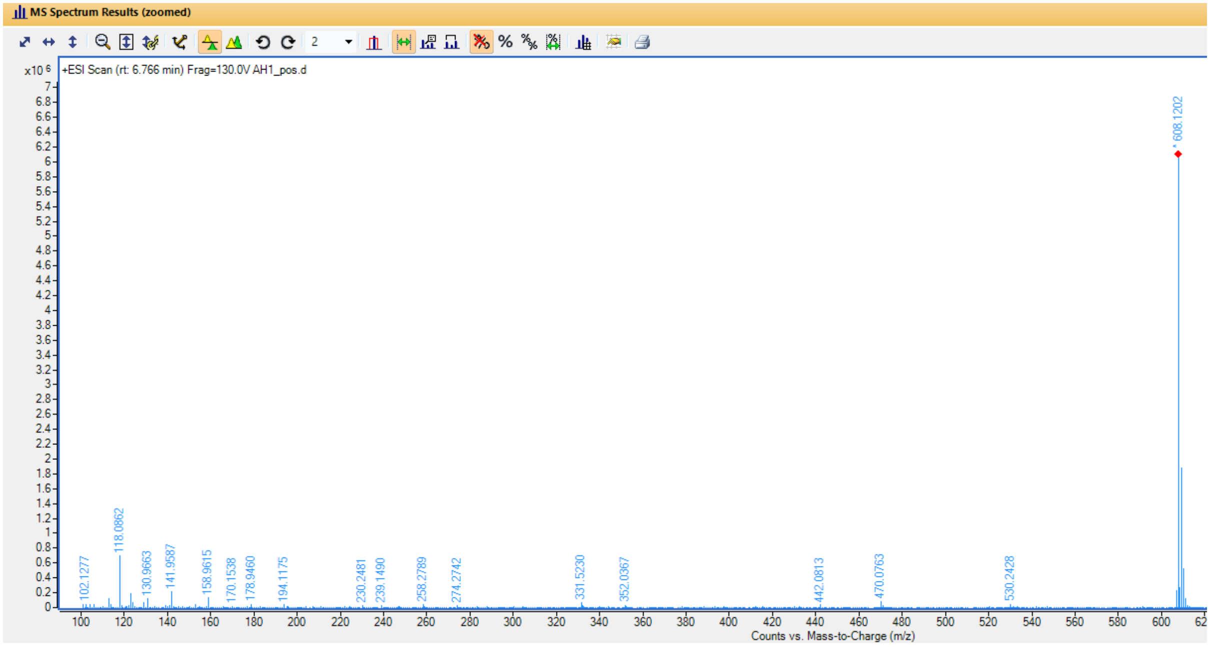

Figure S27. HRMS of compound **7i** in DMSO-d_6 at 75 MHz.

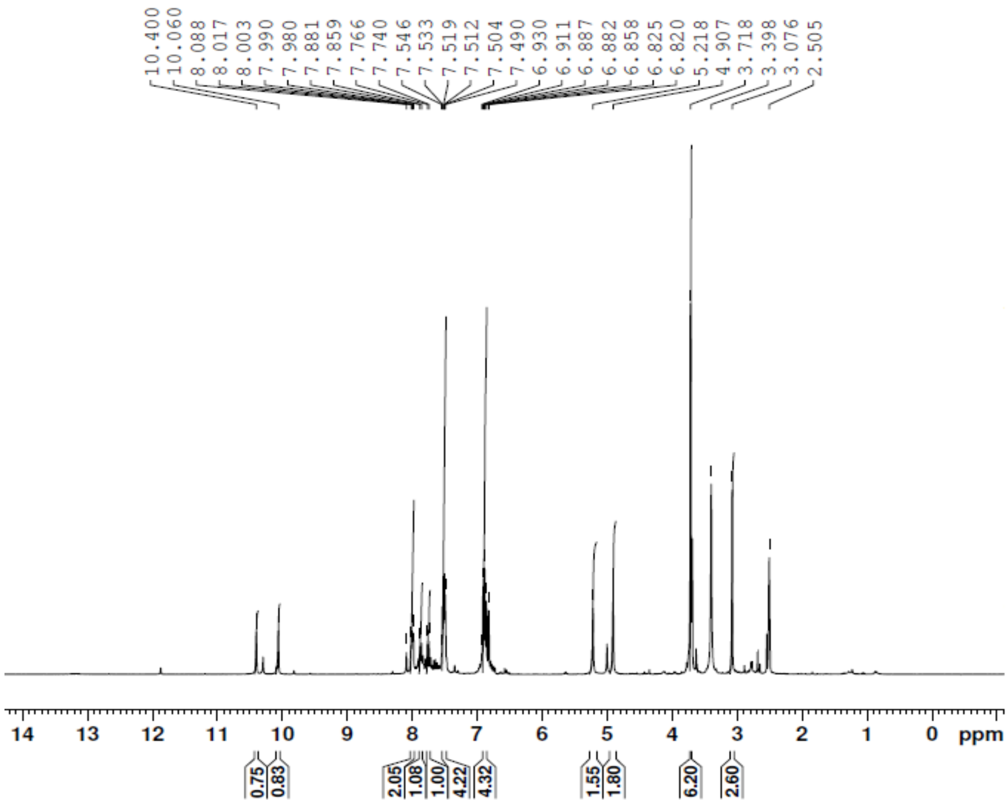

Figure S28. ^{1}H NMR of compound **7j** in DMSO-d_6 at 300 MHz.

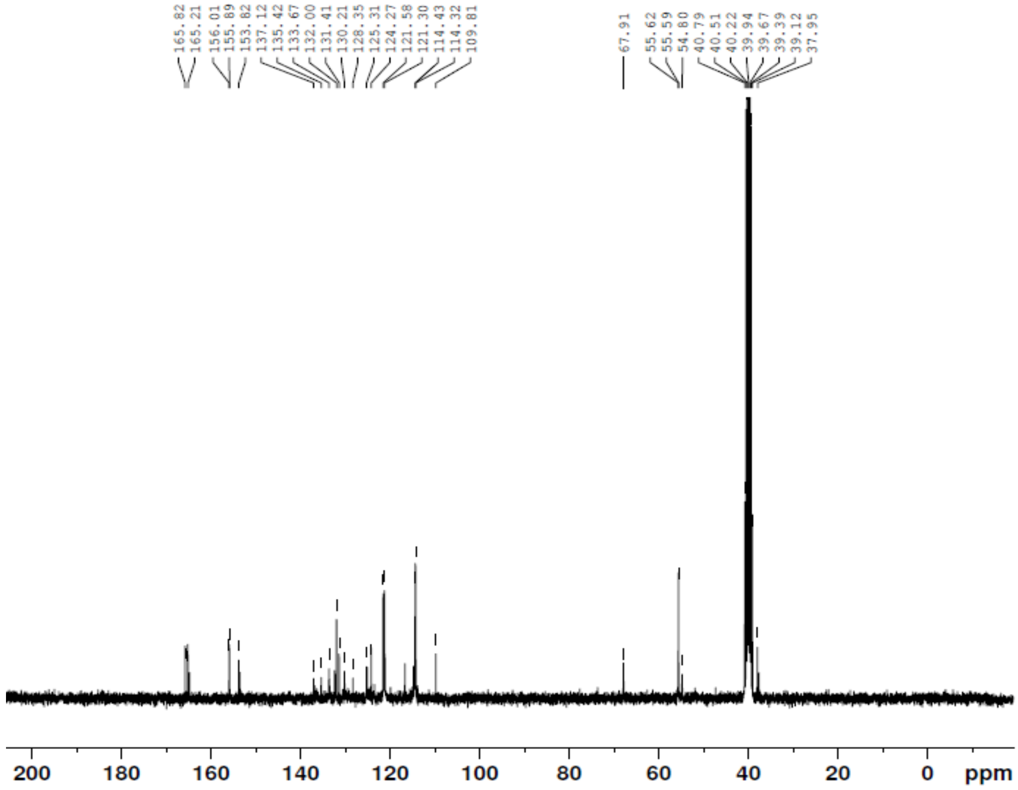

Figure S29. ^{13}C NMR of compound **7j** in DMSO-d_6 at 75 MHz.

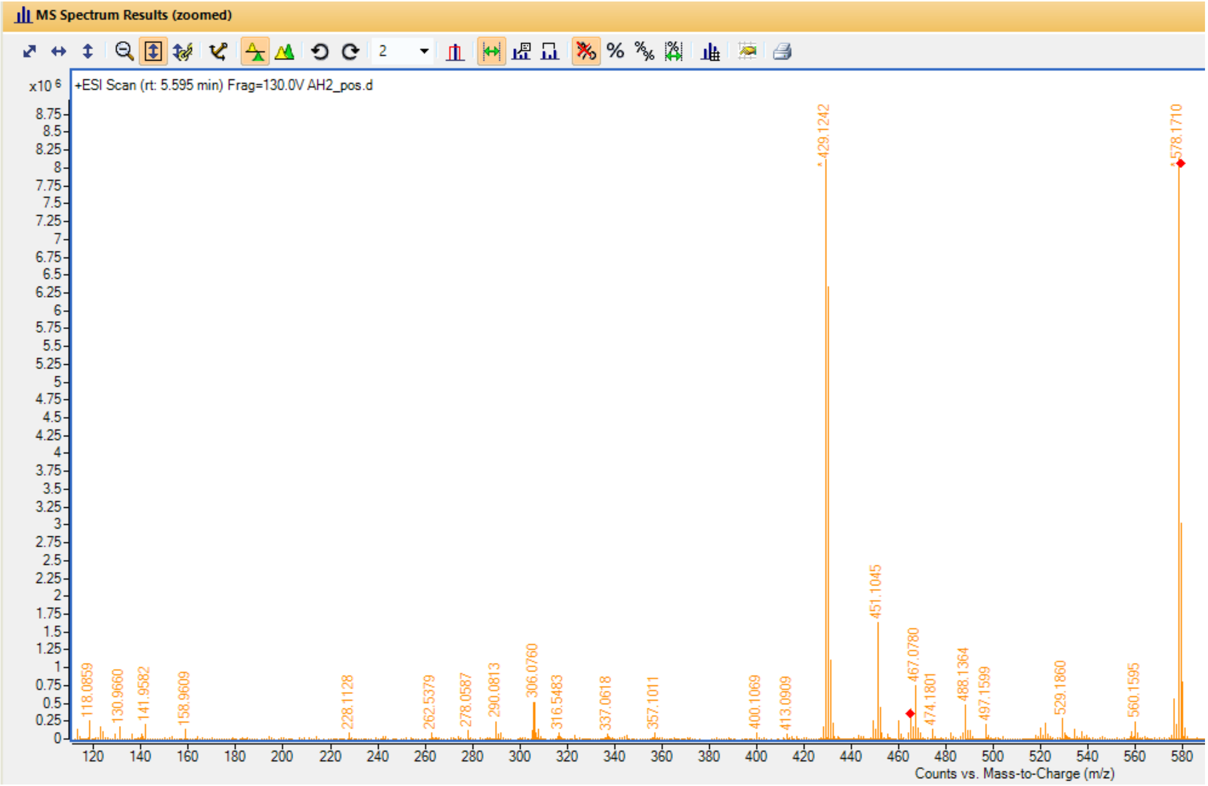

Figure S30. HRMS of compound **7j**.

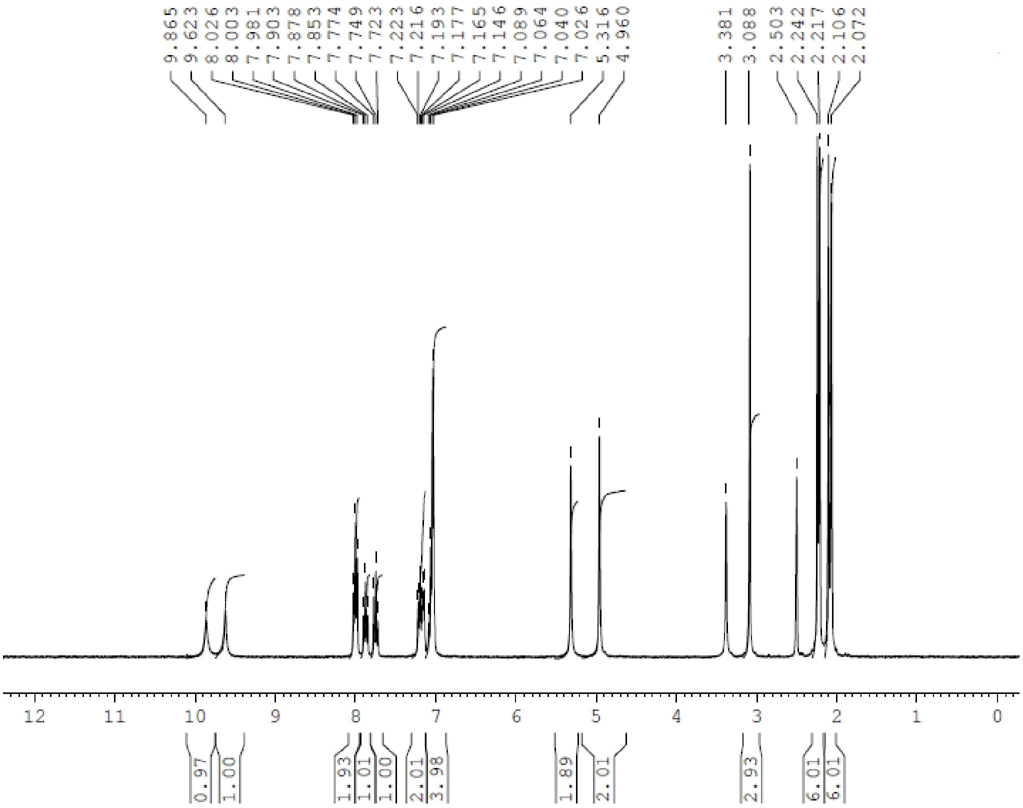

Figure S31. ^{1}H NMR of compound **7k** in DMSO-d$_6$ at 300 MHz.

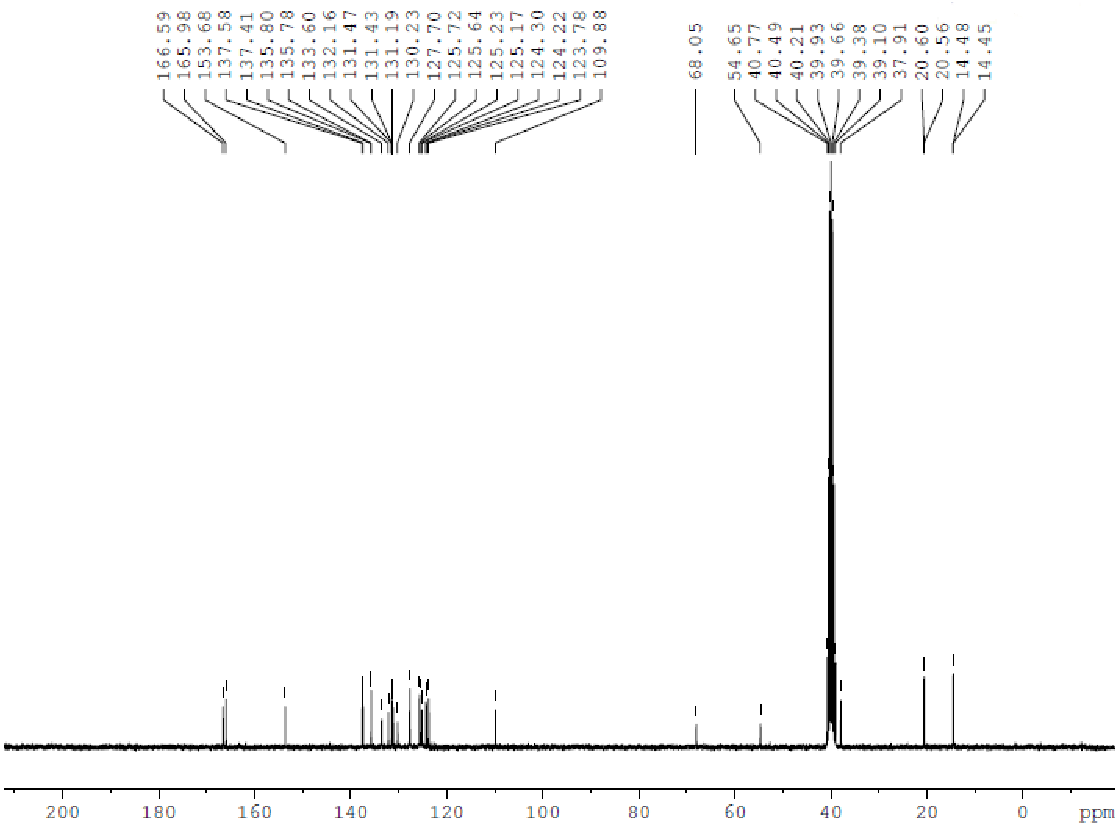

Figure S32. ^{13}C NMR of compound **7k** in DMSO-d$_6$ at 75 MHz.

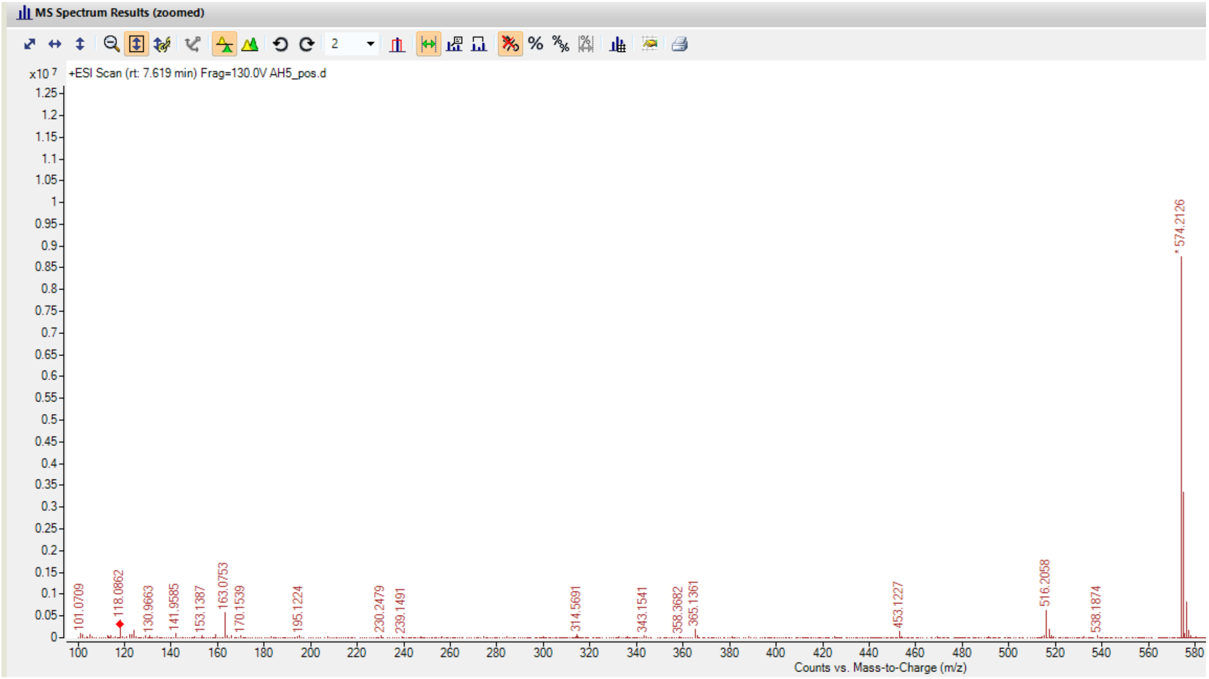

Figure S33. HRMS of compound **7k**.

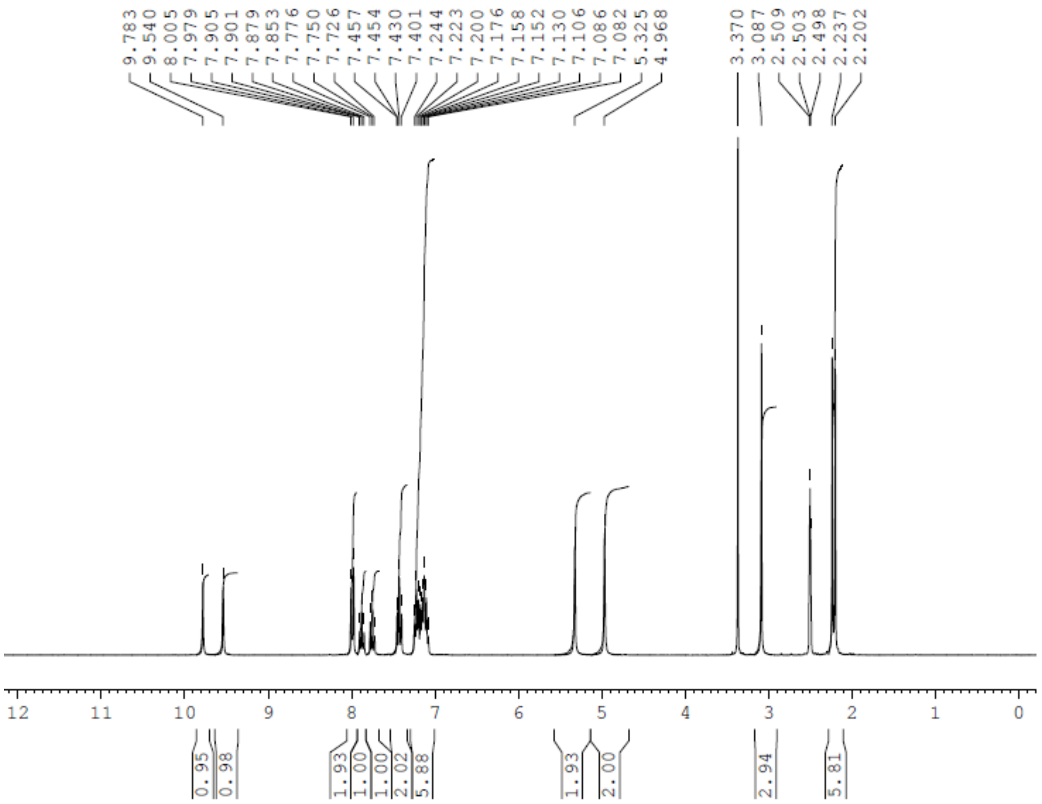

Figure S34. ^{1}H NMR of compound **7l** in DMSO-d_6 at 300 MHz.

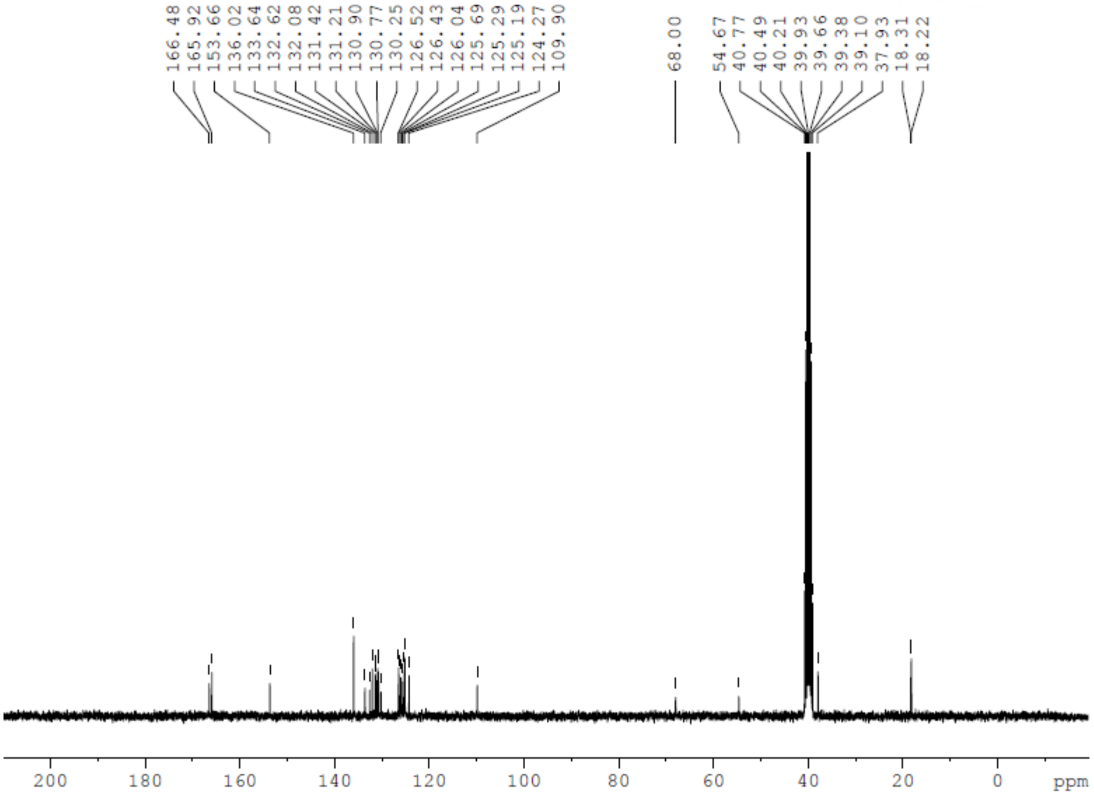

Figure S35. ^{13}C NMR of compound **7l** in DMSO-d_6 at 75 MHz.

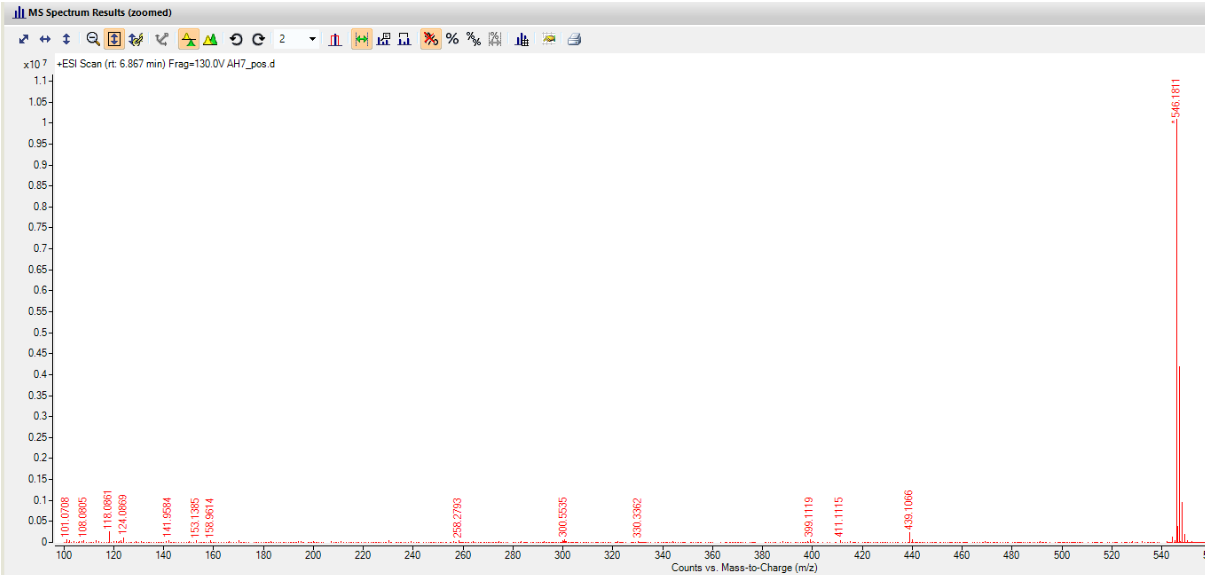

Figure S36. HRMS of compound **7l**.

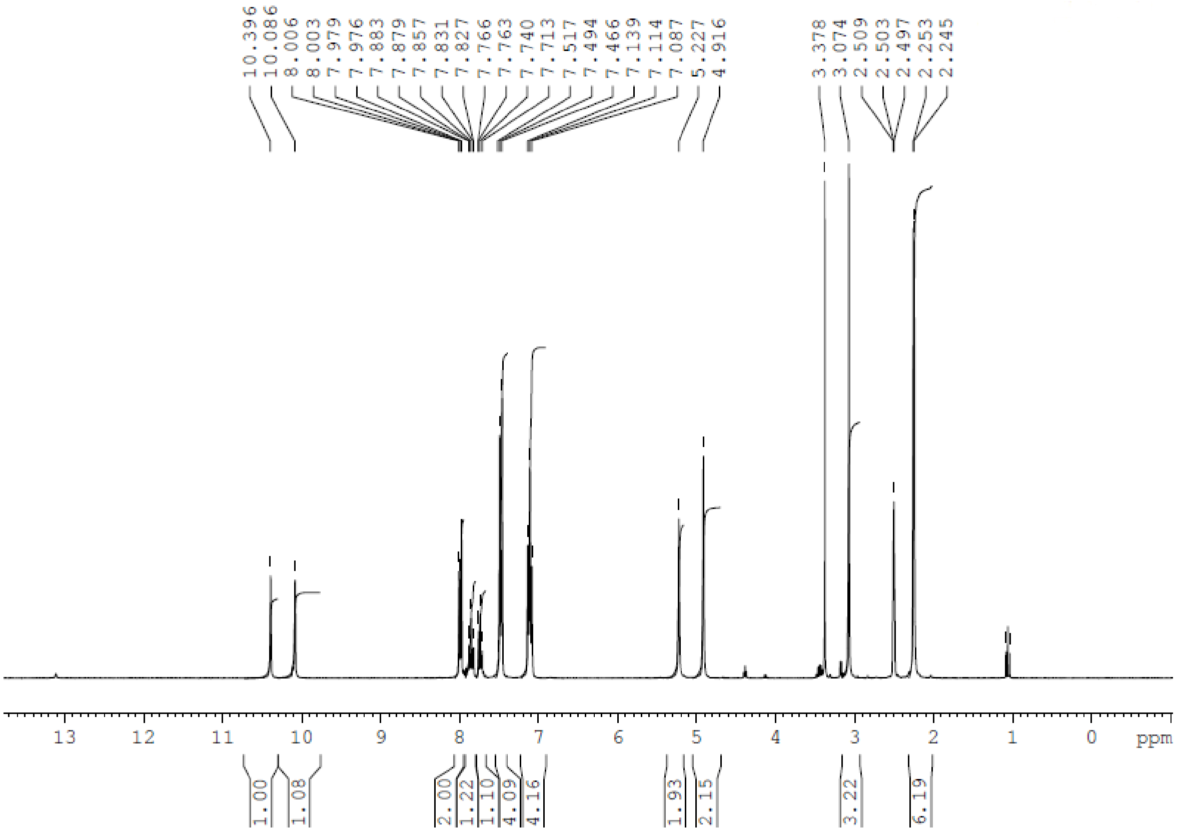

Figure S37. ^{1}H NMR of compound **7m** in DMSO-d_6 at 300 MHz.

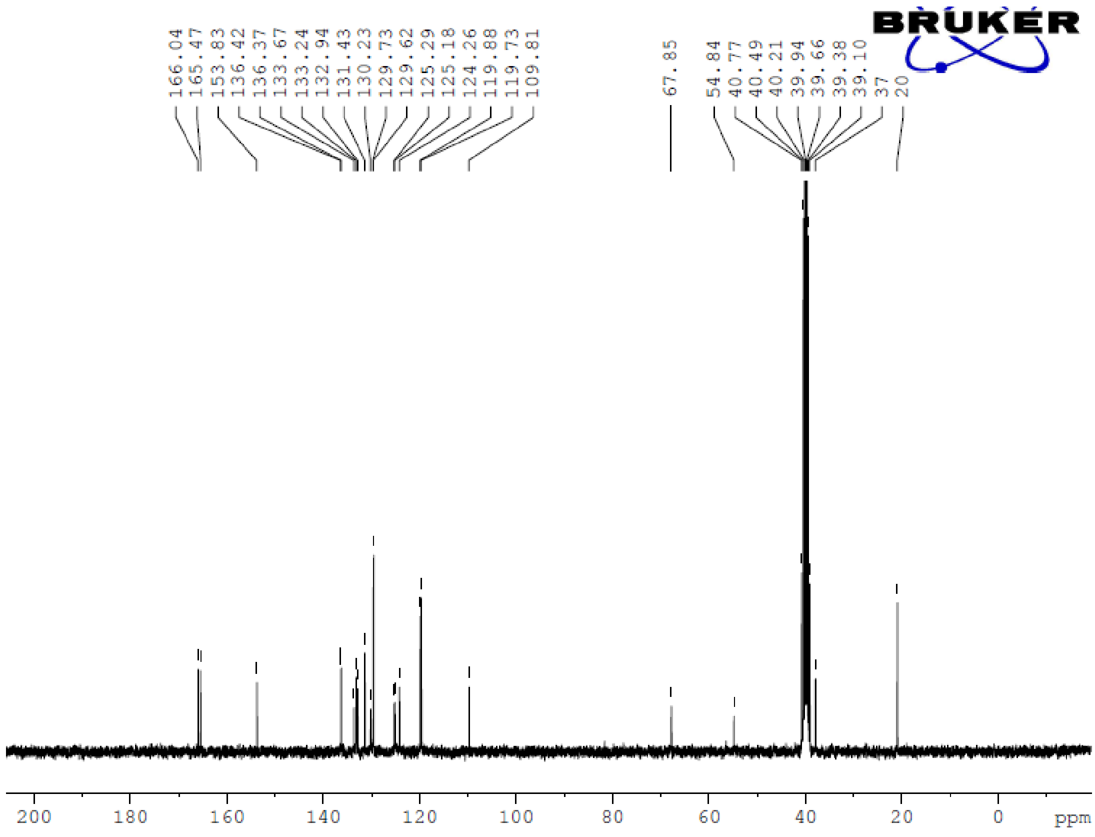

Figure S38. ^{13}C NMR of compound **7m** in DMSO-d$_6$ at 75 MHz.

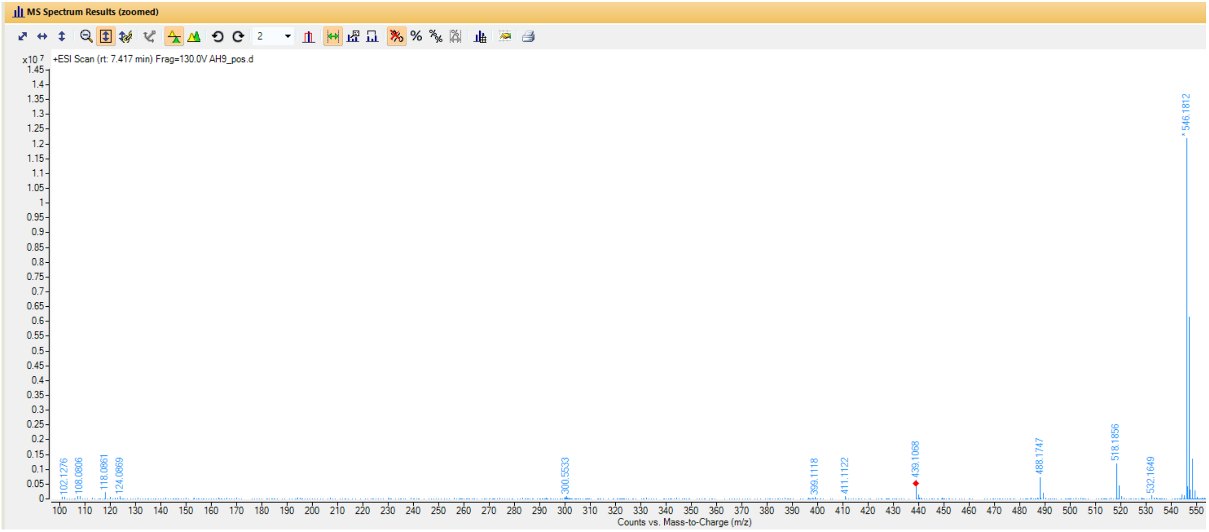

Figure S39. HRMS of compound **7m**.

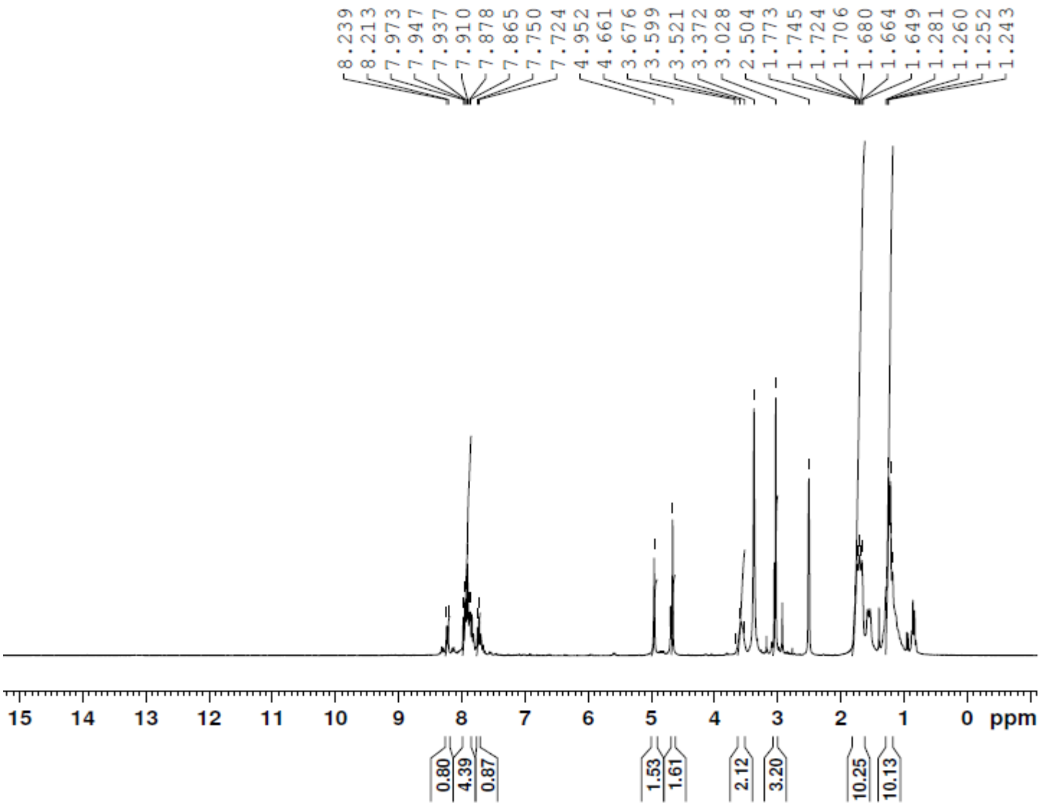

Figure S40. ^{1}H NMR of compound **7n** in DMSO-d$_6$ at 300 MHz.

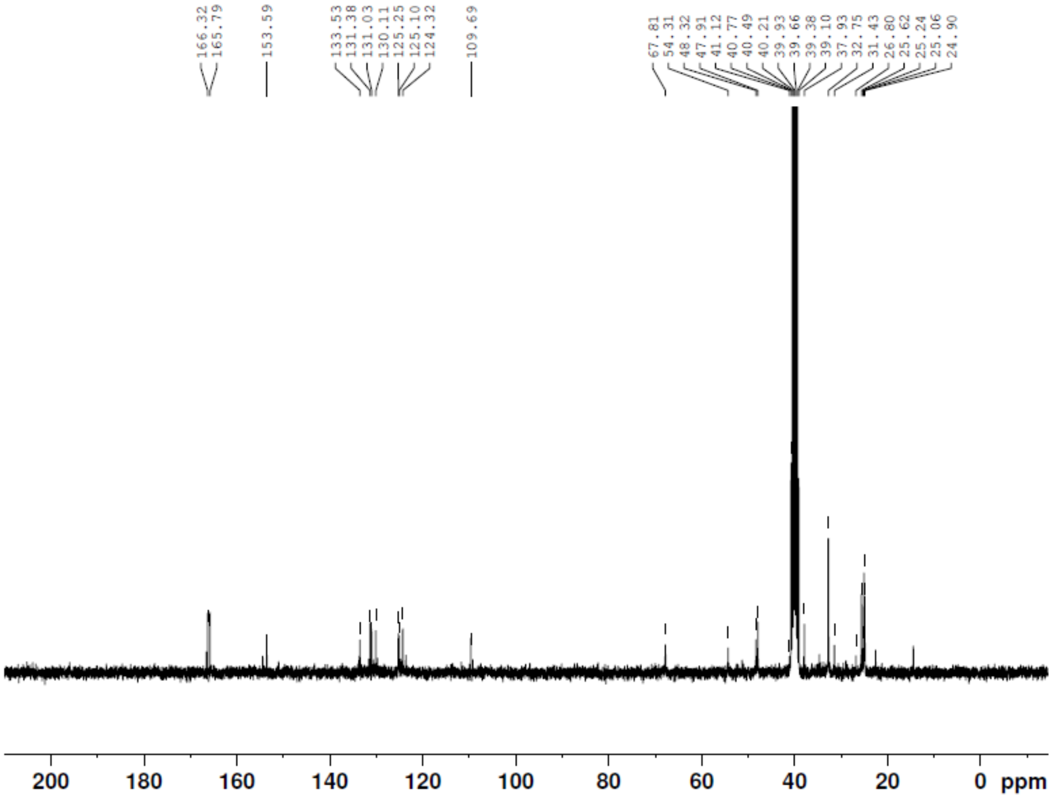

Figure S41. ^{13}C NMR of compound **7n** in DMSO-d$_6$ at 75 MHz.

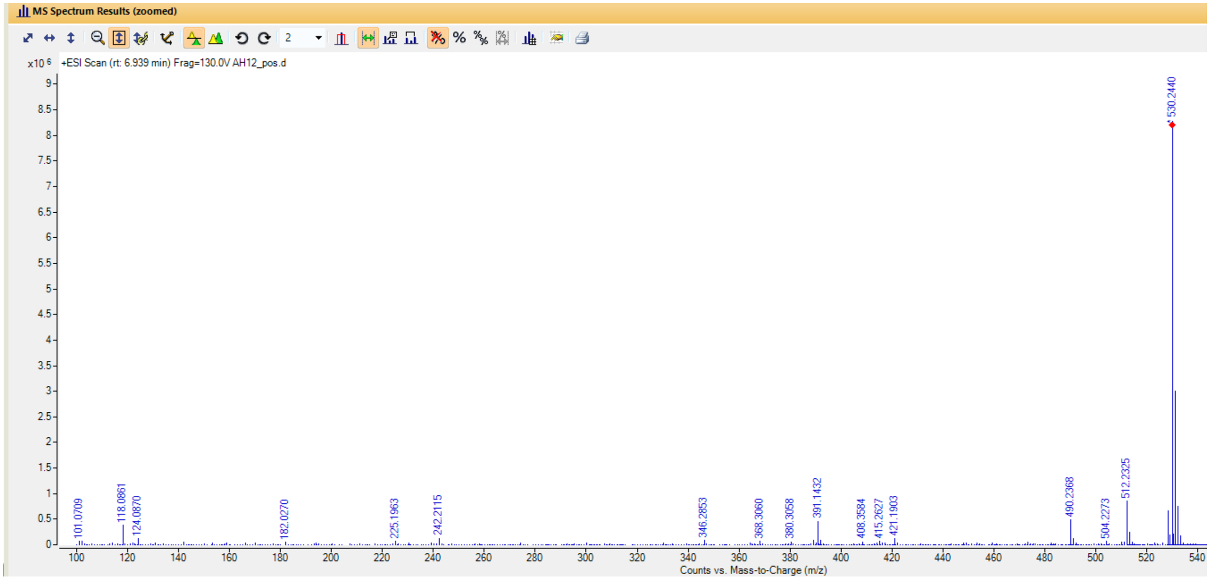

Figure S42. HRMS of compound **7n**.

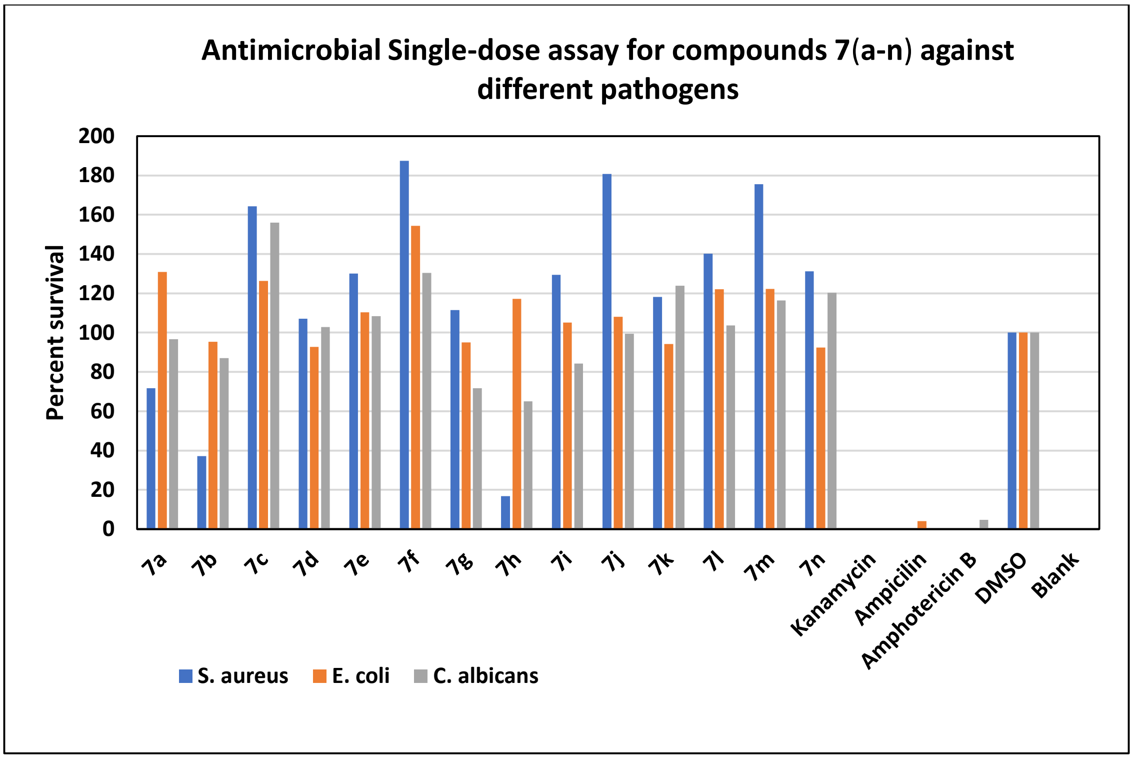

Figure S43: Graph showing results of antimicrobial single-dose microbroth assays of compounds **7a–n**, dose of tested compounds = 125 µg/mL, control antibiotics used: kanamycin = 50 µg/mL (*Staphylococcus aureus* ATCC 25923), ampicillin = 50 µg/mL (*Escherichia coli* ATCC 8739), and amphotericin B = 25 µg/mL (*Candida albicans* ATCC 90027). Dimethylsulfoxide (DMSO) was used as the negative control at 1.25% v/v.

Table S1: Results of single-dose antimicrobial and cytotoxic assays for compounds **7a**–**n**; dosage of tested compounds = 125 µg/mL; antibiotic control dosage (kanamycin = 50 µg/mL (*Staphylococcus aureus* ATCC 25923), ampicillin = 50 µg/mL (*Escherichia coli* ATCC 8739), and amphotericin B = 25 µg/mL (*Candida albicans* ATCC 90027). For cytotoxic assays, dosage of tested compounds = 10 µM; dosage of mensacarcin = 10 µM.

**Samples**	**Percent survival / % Cell viability**			
	***S. aureus* (SD)**	***E.coli* (SD)**	***C. albicans* (SD)**	**HCT-116 (SD)**
**7a**	71.75 (21)	>100	96.68 (12)	85.40 (3)
**7b**	**37.17 (13)**	95.34 (16)	86.98 (6)	88.90 (3)
**7c**	>100	>100	>100	81.40 (2)
**7d**	>100	92.68 (2)	>100	>100
**7e**	>100	>100	>100	>100
**7f**	>100	>100	>100	>100
**7g**	>100	95.01 (1)	71.75 (0)	>100
**7h**	**16.73 (3)**	>100	65.10 (5)	>100
**7i**	>100	>100	84.21 (5)	87.30 (6)
**7j**	>100	>100	99.45 (6)	>100
**7k**	>100	94.18 (0.1)	>100	90.70 (4)
**7l**	>100	>100	>100	**63.60 (1)**
**7m**	>100	>100	>100	>100
**7n**	>100	92.35 (1)	>100	>100
Kanamycin	0.37 (0.7)	–	–	–
Ampicillin	–	4.16 (3)	–	–
Amphotericin B	–	–	4.71 (1)	
Mensacarcin	–	–	–	11.50 (1)

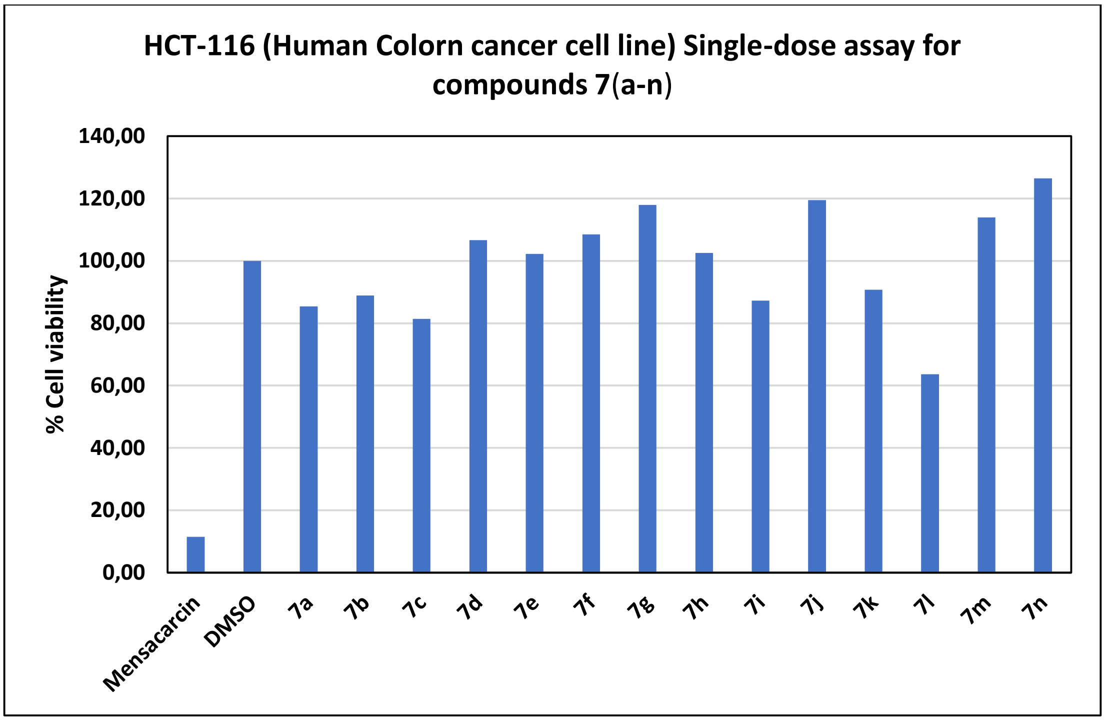

Figure S44: Graph showing cell viability of compounds **7a–n** against human colon carcinoma cell line (HCT-116, ATCC CCL-247), dose of tested compounds = 10 μM, dose of positive control (mensacarcin) = 10 μM.

3. Characterization data of compounds 7a–n

All the synthesized compounds in DMSO-d_6 were scanned for their ^{1}H NMR spectra at 300 MHz and ^{13}C NMR at 75 MHz.

***N*-(4-Chlorophenyl)-2-(3-hydroxy-4-methyl-5,5-dioxidobenzo[*e*]pyrazolo [4,3-*c*] [1,2] thiazine-1(4*H*)-yl)acetamide (7a)**

Off white solid, Yield 335 mg (80%), m.p 156-158 ºC, FT-IR: 1537 (C=O), 1335, 1171 (SO_2), 1239 (C–N); ^{1}H NMR (δ): *N*-CH_3 (linked with thiazine ring) = 3.08 (s, 3H), *N*-CH_2 (linked with pyrazole ring) = 4.95 (s, 2H), N-H (amide) = 10.36 (s, 1H), O-H (linked with pyrazole ring) = 13.12 (s, 1H); Aromatic protons: 7.37 (d, 2H, J = 8.8 Hz), 7.65 (d, 2H, J = 8.7 Hz), 7.71 (d, 1H, J = 6.0 Hz), 7.86 (t, 1H, J = 7.5 Hz), 7.92 (t, 2H, J = 7.6 Hz); ^{13}C NMR (δ): 38.1, 67.8, 109.2, 121.3, 123.7, 124.4, 124.8, 127.5, 129.2, 130.0, 130.2, 130.6, 133.8, 138.0, 154.3, 166.9; HR-MS: Mass to charge ratio calculated from theoretical formula $C_{18}H_{15}ClN_4O_4S$ $[M+H]^+$ 419.0575, observed 419.0578, Δppm 0.71.

***N*-(3-Chlorophenyl)-2-(3-hydroxy-4-methyl-5,5-dioxidobenzo[*e*] pyrazolo [4,3-*c*] [1,2] thiazine-1(4*H*)-yl)acetamide (7b)**

Peach colored solid, Yield 331 mg (79%), m.p 170-172 ºC, FT-IR: 1592 (C=O), 1335, 1179 (SO_2), 1298 (C–N); ^{1}H NMR (δ): *N*-CH_3 (linked with thiazine ring) = 3.08 (s,3H), *N*-CH_2 (linked with pyrazole ring) = 4.96 (s, 2H), N-H (amide) = 10.45 (s, 1H), O-H (linked with pyrazole ring) = 13.21 (s, 1H); Aromatic protons: 7.13 (d, 1H, J = 6.9 Hz), 7.35 (t, 1H, J = 8.1 Hz), 7.48 (d, 1H, J = 8.4 Hz), 7.61 (t, 1H, J = 7.7 Hz), 7.70 (t, 1H, J = 6.8 Hz), 7.83 (1H, s), 7.93 (d, 2H, J = 8.1 Hz); ^{13}C NMR (δ): 38.1, 67.8, 109.1, 118.1, 119.2, 123.4, 123.7, 124.5, 124.7, 124.8, 130.2, 130.6, 131.0, 133.6, 133.7, 140.5, 154.2, 167.1; HR-MS: Mass to charge ratio calculated from theoretical formula $C_{18}H_{15}ClN_4O_4S$ $[M+H]^+$ 419.0575 and observed 419.0578, Δppm 0.71.

***N*-(3,5-Dimethylphenyl)-2-(3-hydroxy-4-methyl-5,5-dioxidobenzo[*e*]pyrazolo[4,3-*c*][1,2]thiazine-1(4*H*)-yl)acetamide (7c)**

Light brown solid, Yield 341 mg (83%), m.p 152-154 ºC, FT-IR: 1510 (C=O), 1339, 1158 (SO_2), 1266 (C–N); ^{1}H NMR (δ): Ar-CH_3 (linked with *N*-aryl-acetamide) = 2.23 (s, 6H), *N*-CH_3 (linked with thiazine ring) = 3.09 (s, 3H), *N*-CH_2 (linked with pyrazole ring) = 4.92 (s, 2H), N-H (amide) = 10.04 (s, 1H), O-H (linked with pyrazole ring) = 13.10 (s,

1H); Aromatic protons: 6.70 (s, 1H), 7.24 (s, 2H), 7.69 (t, 1H, *J* = 7.5 Hz), 7.86 (t, 1H, *J* = 7.4 Hz), 7.89 – 7.96 (m, 2H); ^{13}C NMR (δ): 21.5, 38.1, 67.7, 109.2, 117.5, 123.7, 124.4, 124.8, 125.5, 129.9, 130.2, 130.6, 133.7, 138.2, 138.9, 154.4, 166.5; HR-MS: Mass to charge ratio calculated from theoretical formula $C_{20}H_{20}N_4O_4S$ $[M+H]^+$ 413.1278 and observed 413.1289, Δppm 2.66.

2-(3-Hydroxy-4-methyl-5,5-dioxidobenzo[e]pyrazolo[4,3-*c*][1,2] thiazine-1(4*H*)-yl)-*N*-(*m*-tolyl)acetamide (7d)

Light orange solid, Yield 322 g (81%), m.p 155-156 ºC, FT-IR: 1537 (C=O), 1336, 1155 (SO_2), 1260 (C–N); ^{1}H NMR (δ): Ar-CH_3 (linked with *N*-aryl-acetamide) = 2.27 (s, 3H), *N*-CH_3 (linked with thiazine ring) = 3.09 (s, 3H), *N*-CH_2 (linked with pyrazole ring) = 4.94 (s, 2H), N-H (amide) = 10.14 (s, 1H), O-H (linked with pyrazole ring) = 13.14 (s, 1H); Aromatic protons: 6.88 (d, 1H, *J* = 7.5 Hz), 7.19 (t, 1H, *J* = 7.8 Hz), 7.39 (d, 1H, *J* = 8.3 Hz), 7.48 (s, 1H), 7.70 (t, 1H, *J* = 7.5 Hz), 7.80 – 8.04 (m, 3H); ^{13}C NMR (δ): 21.6, 38.1, 67.8, 109.1, 117.0, 120.2, 123.7, 124.5, 124.6, 124.8, 129.1, 130.0, 130.2, 130.6, 133.7, 138.5, 139.0, 154.3, 166.6.; HR-MS: Mass to charge ratio calculated from theoretical formula $C_{19}H_{18}N_4O_4S$ $[M+H]^+$ 399.1122 and observed 399.1128, Δppm 1.50.

2-(3-Hydroxy-4-methyl-5,5-dioxidobenzo[e]pyrazolo[4,3-*c*][1,2] thiazine-1(*4H*)-yl)-*N*-(2-nitrophenyl)acetamide (7e)

Yellow solid, Yield 334 g (78%), m.p 151-153 ºC, FT-IR: 1494 (C=O), 1335, 1155 (SO_2), 1248 (C–N); ^{1}H NMR (δ): *N*-CH_3 (linked with thiazine ring) = 3.11 (s, 3H), *N*-CH_2 (linked with pyrazole ring) = 5.00 (s, 2H), N-H (amide) = 10.75 (s, 1H), O-H (linked with pyrazole ring) = 13.23 (s, 1H); Aromatic protons: 7.38 (t, 1H, *J* = 7.8 Hz), 7.71 (t, 1H, *J* = 7.5 Hz), 7.77 (t, 1H, *J* = 7.8 Hz), 7.87 (t, 1H, *J* = 7.4 Hz), 7.93 (t, 2H, *J* = 6.5 Hz), 8.10 (t, 2H, *J* = 8.9 Hz); ^{13}C NMR (δ): 38.2, 68.0, 109.1, 123.8, 124.4, 124.5, 124.8, 125.5, 125.8, 130.2, 130.3, 130.7, 132.0, 133.8, 135.4, 140.6, 153.9, 167.30; HR-MS: Mass to charge ratio calculated from theoretical formula $C_{18}H_{15}N_5O_6S$ $[M+H]^+$ 430.0816 and observed 430.0821, Δppm 1.16.

***N*-Benzyl-2-(3-hydroxy-4-methyl-5,5-dioxidobenzo[*e*]pyrazolo[4,3-*c*][1,2] thiazine-1 (4*H*)-yl)acetamide (7f)**

White solid, Yield 308 mg (77%), m.p 154-156 ºC, FT-IR: 1536 (C=O), 1336, 1153 (SO_2), 1228 (C–N); ^{1}H NMR (δ): *N*-CH_3 (linked with thiazine ring) = 3.06 (s, 3H), Ar-CH_2 (benzyl) = 4.35 (d, *J* = 2.9 Hz, 2H), *N*-CH_2 (linked with pyrazole ring) = 4.80 (s, 2H), N-H (amide) = 8.65 (s, 1H), O-H (linked with pyrazole ring) = 13.14 (s, 1H); Aromatic protons: 7.13 – 7.48 (m, 5H), 7.70 (t, 1H, *J* = 7.2 Hz), 7.90 (t, 3H, *J* = 11.4 Hz); ^{13}C NMR (δ): 38.1, 42.2, 67.9, 109.1, 123.7, 124.5, 124.7, 127.2, 127.6, 128.7, 129.9, 130.2, 130.6, 133.7, 139.7, 154.3, 167.91; HR-MS: Mass to charge ratio calculated from theoretical formula $C_{19}H_{18}N_4O_4S$ $[M+H]^+$ 399.1122 and observed 399.1131, Δppm 2.25.

2-(3-Hydroxy-4-methyl-5,5-dioxidobenzo[*e*]pyrazolo[4,3-*c*][1,2] thiazine-1(4*H*)-yl)-*N*-(naphthalen-1-yl)acetamide (7g)

Light orange solid, Yield 351 mg (81%), m.p 161-163 ºC, FT-IR: 1502 (C=O), 1338, 1157 (SO_2), 1252 (C–N); ^{1}H NMR (δ): *N*-CH_3 (linked with thiazine ring) = 3.11 (s, 3H), *N*-CH_2 (linked with pyrazole ring) = 5.13 (s, 2H), N-H (amide) = 10.22 (s, 1H), O-H (linked with pyrazole ring) = 13.20 (s, 1H); Aromatic protons: 7.50 (d, 1H, *J* = 7.8 Hz), 7.51 – 7.60 (m, 2H), 7.63 – 7.76 (m, 2H), 7.80 (d, 1H, *J* = 8.2 Hz), 7.87 (t, 1H, *J* = 7.6 Hz), 7.95 (d, 3H, *J* = 8.0 Hz), 8.08 – 8.13 (m, 1H); ^{13}C NMR (δ): 38.1, 68.0, 109.2, 122.6, 123.4, 123.7, 124.5, 124.8, 126.0, 126.2, 126.4, 126.6, 128.6, 130.0, 130.2, 130.7, 133.4, 133.8, 134.2, 143.4, 154.5, 167.6; HR-MS: Mass to charge ratio calculated from theoretical formula $C_{22}H_{18}N_4O_4S$ $[M+H]^+$ 435.1122 and observed 435.1128, Δppm 1.37.

***N*-(4-Bromophenyl)-2-(3-hydroxy-4-methyl-5, 5-dioxidobenzo [*e*] pyrazolo[4,3-*c*][1,2] thiazine-1(4*H*)-yl)acetamide (7h)**

Light yellow solid, Yield 378 mg (82%), m.p 185-186 ºC, FT-IR: 1538 (C=O), 1349, 1172 (SO_2), 1270 (C–N); ^{1}H NMR (δ): *N*-CH_3 (linked with thiazine ring) = 3.08 (s, 3H), *N*-CH_2 (linked with pyrazole ring) = 4.95 (s, 2H), N-H (amide) = 10.40 (s, 1H), O-H (linked with pyrazole ring) = 13.03 (s, 1H); Aromatic protons: 7.50 (d, 2H, *J* = 8.9 Hz), 7.60 (d, 2H, *J* = 8.9 Hz), 7.69 (t, 1H, *J* = 7.5 Hz), 7.85 (t, 1H, *J* = 7.7 Hz), 7.93 (d, 2H, *J* = 9.0 Hz); ^{13}C NMR (δ): 38.2, 67.8, 109.1, 115.5, 121.7, 123.7, 124.6, 124.8, 130.1,

130.6, 132.1, 133.7, 138.4, 154.3, 166.9; HR-MS: Mass to charge ratio calculated from theoretical formula $C_{18}H_{15}BrN_4O_4S$ $[M+H]^+$ 463.0078 and observed 463.0070, Δppm -1.72.

2-((4-Methyl-1-(2-((4-nitrophenyl)amino)-2-oxoethyl)-5,5-dioxido-1,4-dihydro benzo[*e*] pyrazolo[4,3-*c*][1,2]thiazine-3-yl)oxy)-*N*-(4-nitrophenyl) acetamide (7i)

Light orange brown solid, Yield 475 mg (78%), m.p 185-187 ºC, FT-IR: 1602, 1504 (C=O), 1328, 1175 (SO_2), 1253 (C–N); ^1H NMR (δ): *N*-CH_3 (linked with thiazine ring) = 3.08 (s, 3H), *N*-CH_2 (linked with pyrazole ring) = 5.01 (s, 2H), *O*-CH_2 (linked with pyrazole ring) = 5.31 (s, 2H), N-H (amide) = 10.82 (s, 1H), N-H (amide) = 11.07 (s, 1H); Aromatic protons: 7.82-8.20 (m, 12H); ^{13}C NMR (δ): 37.9, 55.0, 68.2, 112.6, 119.5, 125.5, 127.6, 129.1, 130.5, 131.8, 133.7, 134.7, 143.3, 145.1, 166.7, 166.7; HR-MS: Mass to charge ratio calculated from theoretical formula $C_{26}H_{21}N_7O_9S$ $[M+H]^+$ 608.1194 and observed 608.1202, Δppm 1.31.

***N*-(4-Methoxyphenyl)-2-(3-(2-((4-methoxyphenyl)amino)-2-oxoethoxy)-4-methyl-5,5-dioxidobenzo[*e*]pyrazolo[4,3-*c*][1,2]thiazine-1(4*H*)-yl)acetamide (7j)**

Light orange solid, Yield 442 mg (77%), m.p 180-182 ºC, FT-IR: 1610, 1506 (C=O), 1334, 1170 (SO_2), 1232 (C–N); ^1H NMR (δ): *N*-CH_3 (linked with thiazine ring) = 3.08 (s, 3H), *O*-CH_3 (linked with *N*-aryl-acetamide) = 3.72 (s, 6H), *N*-CH_2 (linked with pyrazole ring) = 4.91(s, 2H), *O*-CH_2 (linked with pyrazole ring) = 5.22 (s, 2H), N-H (amide) = 10.06 (s, 1H), N-H (amide) = 10.40 (s, 1H); Aromatic protons: 6.82-6.93 (m, 4H), 7.49-7.54 (m, 4H), 7.75 (d, 1H, *J* = 7.8 Hz), 7.87 (d, 1H, *J* = 6.8 Hz), 7.98-8.08 (m, 2H); ^{13}C NMR (δ): 38.0, 54.8, 55.6, 55.6, 67.9, 109.9, 114.3, 114.4, 121.3, 121.6, 124.3, 125.3, 128.4, 130.2, 131.4, 132.0, 133.7, 135.4, 137.1, 153.8, 155.9, 156.0, 165.2, 165.8; HR-MS: Mass to charge ratio calculated from theoretical formula $C_{28}H_{27}N_5O_7S$ $[M+H]^+$ 578.1704 and observed 578.1710, Δppm 1.03.

***N*-(2,3-Dimethylphenyl)-2-(3-(2-((2,3-dimethylphenyl)amino)-2-oxoethoxy)-4-methyl-5,5-dioxidobenzo[*e*]pyrazolo[4,3-*c*][1,2]thiazine-1(4*H*)-yl)acetamide (7k)**

Light pink solid, Yield 482 mg (84%), m.p 198-199 ºC, FT-IR: 1670, 1536 (C=O), 1348, 1155 (SO_2), 1268 (C–N); ^1H NMR (δ): Ar-CH_3 (linked with *N*-aryl-acetamide) = 2.07 (s, 3H), Ar-CH_3 (linked with *N*-aryl-acetamide) = 2.11 (s, 3H), Ar-CH_3 (linked with *N*-aryl-acetamide) = 2.22 (s, 3H), Ar-CH_3 (linked with *N*-aryl-acetamide) = 2.24 (s, 3H), *N*-

CH_3 (linked with thiazine ring) = 3.09 (s, 3H), *N*-CH_2 (linked with pyrazole ring) = 4.96 (s, 2H), *O*-CH_2 (linked with pyrazole ring) = 5.32 (s, 2H), N-H (amide) = 9.62 (s, 1H), N-H (amide) = 9.86 (s, 1H); Aromatic protons: 7.00 – 7.10 (m, 4H), 7.18 (ddd, 2H, *J* = 14.2, 6.3, 2.8 Hz), 7.75 (t, 1H, *J* = 7.7 Hz), 7.88 (t, 1H, *J* = 7.4 Hz), 8.00 (td, 2H, *J* = 6.3, 3.2 Hz); ^{13}C NMR (δ): 14.4, 14.5, 20.6, 20.6, 37.9, 54.6, 68.1, 109.9, 123.8, 124.2, 124.3, 125.2, 125.2, 125.6, 125.7, 127.7, 130.2, 131.2, 131.4, 131.5, 132.2, 133.6, 135.8, 135.8, 137.4, 137.6, 153.7, 165.9, 166.6; HR-MS: Mass to charge ratio calculated from theoretical formula $C_{30}H_{31}N_5O_5S$ $[M+H]^+$ 574.2119 and observed 574.2126, Δppm 1.21.

2-((4-Methyl-5,5-dioxido-1-(2-oxo-2-(*o*-tolylamino)ethyl)-1,4-dihydrobenzo [*e*]pyrazolo [4,3-*c*][1,2]thiazine-3-yl)oxy)-*N*-(*o*-tolyl)acetamide (7l)

Light yellow solid , Yield 438 mg (80%), m.p 202-203 ºC, FT-IR: 1610, 1513 (C=O), 1339, 1177 (SO_2), 1260 (C–N); ^1H NMR (δ): Ar-CH_3 (linked with *N*-aryl-acetamide) = 2.20 (s, 3H), Ar-CH_3 (linked with *N*-aryl-acetamide) = 2.24 (s, 3H), *N*-CH_3 (linked with thiazine ring) = 3.09 (s, 3H), *N*-CH_2 (linked with pyrazole ring) = 4.97 (s, 2H), *O*-CH_2 (linked with pyrazole ring) = 5.33 (s, 2H), N-H (amide) = 9.54 (s, 1H), N-H (amide) = 9.78 (s, 1H); Aromatic protons: 7.08 – 7.21 (m, 4H), 7.22 (t, 2H, *J* = 5.4 Hz), 7.43 (t, 2H, *J* = 7.1 Hz), 7.75 (t, 1H, *J* = 7.6 Hz), 7.88 (t, 1H, *J* = 7.8 Hz), 7.99 (d, 2H, *J* = 7.9 Hz); ^{13}C NMR (δ): 18.2, 18.3, 38.0, 54.7, 68.0, 109.9, 124.3, 125.2, 125.3, 125.7, 126.0, 126.4, 126.5, 130.2, 130.8, 130.9, 131.2, 131.4, 132.1, 132.6, 133.6, 136.0, 153.7, 165.9, 166.5; HR-MS: Mass to charge ratio calculated from theoretical formula $C_{28}H_{27}N_5O_5S$ $[M+H]^+$ 546.1806 and observed 546.181, Δppm 0.91.

2-((4-Methyl-5,5-dioxido-1-(2-oxo-2-(*p*-tolylamino)ethyl)-1,4-dihydrobenzo [*e*]pyrazolo [4,3-*c*][1,2]thiazine-3-yl)oxy)-*N*-(*p*-tolyl)acetamide (7m)

Light yellow solid, Yield 431 mg (79%), m.p 205-207 ºC, FT-IR: 1653, 1537 (C=O), 1341, 1180 (SO_2), 1262 (C–N); ^1H NMR (δ): Ar-CH_3 (linked with *N*-aryl-acetamide) = 2.24 (s, 3H), Ar-CH_3 (linked with *N*-aryl-acetamide) = 2.25 (s, 3H), *N*-CH_3 (linked with thiazine ring) = 3.07 (s, 3H), *N*-CH_2 (linked with pyrazole ring) = 4.92 (s, 2H), *O*-CH_2 (linked with pyrazole ring) = 5.23 (s, 2H), N-H (amide) = 10.09 (s, 1H), N-H (amide) = 10.40 (s, 1H); Aromatic protons: 7.05 – 7.17 (m, 4H), 7.48 (d, 2H, *J* = 8.4 Hz), 7.74 (t, 1H, *J* = 8.0 Hz), 7.86 (t, 1H, *J* = 7.7 Hz), 7.99 (d, 2H, *J* = 9.1 Hz); ^{13}C NMR (δ): 21.0, 38.0, 54.8, 67.8, 109.8, 119.7, 119.9, 124.3, 125.2, 125.3, 129.6, 129.7, 130.2, 131.4,

132.9, 133.2, 133.7, 136.4, 136.4, 153.8, 165.5, 166.0; HR-MS: Mass to charge ratio calculated from theoretical formula $C_{28}H_{27}N_5O_5S$ $[M+H]^+$ 546.1806 and observed 546.1812, Δppm 1.09.

***N*-Cyclohexyl-2-(3-(2-(cyclohexylamino)-2-oxoethoxy)-4-methyl-5,5 dioxido benzo[*e*] pyrazolo[4,3-*c*][1,2]thiazine-1(4*H*)-yl)acetamide (7n)**

Light orange solid, Yield 417 mg (79%), m.p 196-198 ºC, FT-IR: 1657, 1511 (C=O), 1341, 1152 (SO_2), 1252 (C–N); ^{1}H NMR (δ): CH_2 (cyclohexyl) = 1.26 – 1.19 (m, 10H), CH_2 (cyclohexyl) = 1.76 – 1.64 (m, 10H), *N*-CH_3 (linked with thiazine ring) = 3.03 (s, 3H), CH (cyclohexyl) = 3.61 – 3.53 (m, 2H), *N*-CH_2 (linked with pyrazole ring) = 4.66 (s, 2H), *O*-CH_2 (linked with pyrazole ring) = 4.95 (s, 2H), N-H (amide) = 7.74 (d, *J* = 7.8 Hz, 1H), N-H (amide) = 8.23 (d, *J* = 7.8 Hz, 1H); Aromatic protons: 7.91 (m, 4H); ^{13}C NMR (δ): 24.9, 25.1, 25.2, 25.6, 26.8, 31.4, 32.8, 37.9, 47.9, 48.3, 54.3, 67.8, 109.7, 124.3, 125.1, 125.2, 130.1, 131.0, 131.4, 133.5, 153.6, 165.8, 166.3; HR-MS: Mass to charge ratio calculated from theoretical formula $C_{26}H_{35}N_5O_5S$ $[M+H]^+$ 530.2432 and observed 529.2340, Δppm 1.51.

References

1. Zia-ur-Rehman, M.; Choudary, J. A.; Ahmad, S. *Bull. Korean Chem. Soc.* **2005**, 26 (11), 1771–1775, doi:10.5012/bkcs.2005.26.11.1771.
2. Siddique, W. S.; Ahmad, S.; Ullah, I.; Malik. A.; *J. Chem. Soc. Pak.* **2006**, 28 (6), 583-589.
3. Ahmad, N.; Zia-ur-Rehman, M.; Siddiqui, H. L.; Ullah, M. F.; Parvez, M. *Eur. J. Med. Chem.* **2011**, 46, 2368–2377. doi:10.1016/j.ejmech.2011.03.020.
4. Sattar, A.; Ur Rehman, A.; Abbasi, M. A.; Siddiqui, S. Z.; Rasool, S.; Khalid, H.; Lodhi, M .A.; Khan, F. A. *Turk. J. Chem.* **2018**, 42, 401–417. doi:10.3906/kim-1706-50.
